# Prevention and early intervention in eating disorders: findings from a rapid review

**DOI:** 10.1186/s40337-023-00758-3

**Published:** 2023-03-10

**Authors:** Eyza Koreshe, Susan Paxton, Jane Miskovic-Wheatley, Emma Bryant, Anvi Le, Danielle Maloney, National Eating Disorder Research Consortium, Phillip Aouad, Sarah Barakat, Robert Boakes, Leah Brennan, Emma Bryant, Susan Byrne, Belinda Caldwell, Shannon Calvert, Bronny Carroll, David Castle, Ian Caterson, Belinda Chelius, Lyn Chiem, Simon Clarke, Janet Conti, Lexi Crouch, Genevieve Dammery, Natasha Dzajkovski, Jasmine Fardouly, Carmen Felicia, John Feneley, Amber-Marie Firriolo, Nasim Foroughi, Mathew Fuller-Tyszkiewicz, Anthea Fursland, Veronica Gonzalez-Arce, Bethanie Gouldthorp, Kelly Griffin, Scott Griffiths, Ashlea Hambleton, Amy Hannigan, Mel Hart, Susan Hart, Phillipa Hay, Ian Hickie, Francis Kay-Lambkin, Ross King, Michael Kohn, Eyza Koreshe, Isabel Krug, Jake Linardon, Randall Long, Amanda Long, Sloane Madden, Sarah Maguire, Danielle Maloney, Peta Marks, Sian McLean, Thy Meddick, Jane Miskovic-Wheatley, Deborah Mitchison, Richard O’Kearney, Shu Hwa Ong, Roger Paterson, Susan Paxton, Melissa Pehlivan, Genevieve Pepin, Andrea Phillipou, Judith Piccone, Rebecca Pinkus, Bronwyn Raykos, Paul Rhodes, Elizabeth Rieger, Sarah-Catherine Rodan, Janice Russell, Haley Russell, Fiona Salter, Susan Sawyer, Beth Shelton, Urvashnee Singh, Sophie Smith, Evelyn Smith, Karen Spielman, Sarah Squire, Juliette Thomson, Stephen Touyz, Ranjani Utpala, Lenny Vartanian, Sabina Vatter, Andrew Wallis, Warren Ward, Sarah Wells, Eleanor Wertheim, Simon Wilksch, Michelle Williams, Stephen Touyz, Sarah Maguire

**Affiliations:** 1 Faculty of Medicine and Health, InsideOut Institute for Eating Disorders, University of Sydney and Sydney Local Health District, Level 2, Charles Perkins Centre (D17), Sydney, NSW 2006 Australia; 2 School of Psychology and Public Health, La Trobe University, Melbourne, Australia; 3 Healthcare Management Advisors, Melbourne, Australia

**Keywords:** Eating disorder(s), Prevention, Early intervention, Risk factor(s), Mental health, Diagnosis

## Abstract

**Background:**

Eating disorders (EDs) are complex psychological disorders, with low rates of detection and early intervention. They can lead to significant mental and physical health complications, especially if intervention is delayed. Given high rates of morbidity and mortality, low treatment uptake, and significant rates of relapse, it is important to examine prevention, early intervention, and early recognition initiatives. The aim of this review is to identify and evaluate literature on preventative and early intervention programs in EDs.

**Methods:**

This paper is one of a series of Rapid Reviews, designed to inform the Australian National Eating Disorders Research and Translation Strategy 2021–2031, funded, and released by the Australian Government. To provide a current and rigorous review, peer-reviewed articles between 2009 and 2021 published in English were searched across three databases: ScienceDirect, PubMed and Ovid/Medline. Priority was given to high-level evidence including meta-analyses, systematic reviews, Randomised Control Trials, and large population studies. Findings from selected studies pertaining to prevention and early intervention in EDs were evaluated and are presented in this review.

**Results:**

In total, 130 studies were identified in the current review, 72% relating to prevention and 28% to early intervention. Most programs were theory-driven and targeted one or more ED risk factors such as thin-ideal internalisation and/or body dissatisfaction. There is reasonable evidence to support prevention programs reducing risk factors, particularly as part of school or university-based programs, with established feasibility and relatively high acceptance among students. There is increasing evidence around the use of technology (to increase dissemination potential) and for use of mindfulness approaches (targeting emotional resilience). Few longitudinal studies assessing incident cases following participation in a prevention program exist.

**Conclusions:**

Although several prevention and early intervention programs have been shown to significantly reduce risk factors, promote symptom recognition, and encourage help-seeking behaviour, most of these studies have been conducted in older adolescent and university aged students, past the age of peak ED onset. One of the most targeted risk factors, body dissatisfaction, is found in girls as young as 6 years old, indicating a need for further research implementing prevention initiatives at younger ages. Follow-up research is limited; thus, the long-term efficacy and effectiveness of studied programs is unknown. Greater attention should be paid to the implementation of prevention and early intervention programs in identified high-risk cohorts or diverse groups, where a more targeted approach may be necessary.

**Supplementary information:**

The online version contains supplementary material available at (10.1186/s40337-023-00758-3).

## Abstract (highlights)

A large proportion of individuals with eating disorders (ED) or ED symptomatology within the community do not seek treatment or professional help. Low rates of detection and early intervention, low treatment uptake, stigma, shame and lack of motivation to seek help can lead to significant physical and psychological impairment. Research has shown preventative and early intervention programs can significantly reduce ED risk factors, increase self-awareness and motivation to seek help and treatment. Greater allocation of time and resources is important to explore the long-term effectiveness and reach of these interventions within this population, to reduce the prevalence of EDs, particularly within individuals at a greater risk. To reduce the duration of untreated illness and increase treatment uptake, extensive efforts are needed to bridge the gap between early symptom recognition and timely access to evidence-based specialist services.

**Supplementary information:**

The online version contains supplementary material available at (10.1186/s40337-023-00758-3).

## Introduction

Eating disorders (EDs) are complex, multifaceted psychological illnesses with high rates of morbidity and mortality, low rates of early detection and intervention, and high rates of relapse [1–4]. Low levels of help-seeking behaviour, low treatment uptake, and high rates of treatment dropout can lead to prolonged illness, poor prognosis, and high health care costs [1, 5–11]. Considering the significant physical and psychological impacts of EDs it is imperative that research interest is directed towards effective and implementable ED prevention and early intervention strategies.

According to the American Institute of Medicine, prevention programs can be classified into three categories: *universal* (i.e., applied to the whole population irrespective of risk, with a primary aim of managing risk factors and reinforcing protective factors), *selective* (i.e., targets a subgroup who have a higher risk than average), and *indicated* (i.e., targets individuals who already exhibit symptoms without meeting a diagnostic criteria) [12]. A recent systematic review concluded that most ED prevention research has been directed towards selective and indicated prevention programs, targeting individuals at higher risk of developing an ED (e.g., adolescent females) and therefore focused on universal prevention [13]. It is important to note, however, that these classifications are not mutually exclusive, with some ED prevention programs using a combination of features from universal, selective, and indicated prevention programs.

Widely reported prevention programs, including cognitive dissonance-based interventions, media literacy-based programs, and multi-risk factor programs such as school-based interventions, frequently aim to target one or more of the modifiable ED risk factors such as body dissatisfaction, dietary restraint, thin-ideal internalisation, perceived pressure to appear thin, or negative affect [13, 14]. Other empowerment-based and mindfulness-based prevention programs are not as specific in targeting ED risk factors—rather they focus on emotional regulation and empowering individuals to make positive behavioural change concerning food, eating and body image [13, 15]. However, few resources have been allocated to long-term implementation and evaluation of these prevention programs, limiting the ability to demonstrate their effectiveness in preventing future ED onset [16].

Low rates of early intervention and help-seeking behaviour have been widely reported in the literature [17, 18] with only 17–31% of individuals in the community meeting ED diagnostic criteria seeking ED-specific treatment [18, 19]. Early recognition and intervention, especially within the first three years of illness, are integral to recovery, with poorer outcomes being associated with delayed intervention [18]. This is especially important for children and adolescents with an early onset of illness who have been found to experience the longest mean duration of untreated illness [20]. The Duration of Untreated ED (DUED) is the time between onset of an eating disorder and first receiving specialist evidence-based care, and is a crucial factor for early intervention [21, 22]. Early intervention aims to target prodromal or at-risk individuals to prevent or delay onset of illness and increase chances of recovery [23]. However, the time from illness onset to first intervention and duration of illness are often conflated in the literature especially for EDs, highlighting the need for more early intervention studies [24]. According to a recent (2020) systematic review, the average DUED ranges from around 2.5 years for Anorexia Nervosa (AN) to about 6 years for Binge Eating Disorder (BED) [21]. Although there is no established critical window for early intervention for EDs, the most effective time period for delivering an intervention has been found to be within the first three years of illness onset for a greater likelihood of recovery, after which outcomes may deteriorate [23, 25]. Early, timely access to intervention and enhanced help-seeking behaviour can shorten DUED, hence helping prevent prolonged illness and unnecessary suffering [8, 25].

This rapid review aims to identify and evaluate the literature on prevention and early intervention programs in EDs, with a focus on preventing ED onset or reducing existing ED pathology by specifically targeting prodromal ED and the first three years of illness. The aim of the rapid review process is to assess recent, relevant, and high-quality research within the focus area, to inform policy and practice.

## Methods

The Australian Government Commonwealth Department of Health funded the InsideOut Institute for Eating Disorders (IOI) to develop the Australian Eating Disorders Research and Translation Strategy 2021–2031 [26] under the Psychological Services for Hard to Reach Groups initiative (ID 4-8MSSLE). The strategy was developed in partnership with state and national stakeholders including clinicians, service providers, researchers, and experts by lived experience (including consumers and families/carers). Developed through a two-year national consultation and collaboration process, the strategy provides the roadmap to establishing EDs as a national research priority and is the first disorder-specific strategy to be developed in consultation with the National Mental Health Commission. To inform the strategy, IOI commissioned Healthcare Management Advisors (HMA) to conduct a series of rapid reviews (RRs) to broadly assess all available peer-reviewed literature on the six DSM-5 listed EDs.

A RR Protocol [27] was utilised to swiftly synthesise evidence in order to guide public policy and decision-making [28]. This approach has been adopted by several leading health organisations including the World Health Organisation [29] and the Canadian Agency for Drugs and Technologies in Health Rapid Response Service [30], to build a strong evidence base in a timely and accelerated manner, without compromising quality. A RR is not designed to be as comprehensive as a systematic review—it is purposive rather than exhaustive and provides actionable evidence to guide health policy [31].

This RR is a narrative synthesis and sought to adhere to the PRISMA guidelines [32]. It is divided by topic area and presented as a series of papers. Three research databases were searched: ScienceDirect, PubMed and Ovid/Medline. To establish a broad understanding of the progress made in the field of EDs, and to capture the largest evidence base on the past 13 years (originally 2009–2019, but expanded to include 2020–2021), the eligibility criteria for included studies into the rapid review were kept broad. Therefore, included studies were published between 2009 and 2021, in English, and conducted within Western healthcare systems or health systems comparable to Australia in terms of structure and resourcing. The initial search and review process were conducted by three reviewers between 5 December 2019 and 16 January 2020. The re-run for the years 2020–2021 was conducted by two reviewers at the end of May 2021.

The RR presented here had a translational research focus with the objective of identifying evidence relevant to developing optimal care pathways. Searches therefore used a Population, Intervention, Comparison, Outcome (PICO) approach to identify literature relating to population impact, prevention and early intervention, treatment, and long-term outcomes. Purposive sampling focused on high-level evidence studies such as: meta-analyses; systematic reviews; moderately sized randomised controlled studies (RCTs) (n > 50); moderately sized controlled-cohort studies (n > 50); and population studies (n > 500). However, the diagnoses Avoidant/Restrictive Food Intake Disorder (ARFID) and Unspecified Feeding or Eating Disorder (UFED) necessitated less stringent eligibility criteria due to a paucity of published articles. As these diagnoses are newly captured in the DSM-5 (released in 2013, within the allocated search timeframe), the evidence base is emerging, and fewer studies have been conducted. Thus, smaller studies (n =  < 20) and narrative reviews were also considered and included. Grey literature, such as clinical or practice guidelines, protocol papers (without results) and Masters’ theses or dissertations, was excluded.

Full methodological details including eligibility criteria, search strategy and terms and data analysis are published in a separate protocol paper (r) due to the broad scope of the RR, which included a total of 1320 studies [33] (see Additional File 1). Data from included studies relating to prevention and early intervention were synthesised and are presented in the current review.

## Results

The RR identified 1327 studies in total, of which 131 studies were categorised in the ‘Prevention and Early Intervention’ category which were based upon the agreed inclusion/exclusion criteria, as described in the Rapid Review Methodology [33]. Of these, 73% (*n* = 96, Additional File 1) related to prevention and 27% (*n* = 35, Additional File 2) to early intervention (within the first three years of symptomatology) in EDs.

A complete list of included studies for this topic including population, intervention types, and outcome measures can be found in Additional File 2. Results are subdivided into two categories: (i) Prevention, and (ii) Early Intervention.

### Prevention

A total of 96 studies examining prevention programs in EDs, were identified. The three major types of prevention programs (universal, selective, and indicated) used a broad range of approaches focused on reducing ED risk factors and symptoms for preventing future ED onset. Based on the commonly cited prevention approaches and for the purposes of this review, these studies are categorised as (i) cognitive dissonance-based prevention, (ii) universal multi-risk factor programs, (iii) media literacy-based prevention, (iv) mindfulness-based prevention, and (v) other prevention programs.

A 2017 systematic review and meta-analysis of 112 studies found Cognitive Dissonance (CD) and Media Literacy (ML) for EDs to be promising preventive approaches spanning universal, selective and indicated interventions [34]. Despite the small to moderate effect sizes observed, some studies reported a significant reduction in risk for up to three years following the intervention [34]. Among the programs, selective prevention programs aimed at high-risk groups (including females and adolescents over the age of 15) showed the strongest evidence for reducing ED symptomatology [34]. However, for interventions targeting thin-ideal internalisation and body dissatisfaction, research suggests that targeting ages of 12–13 years could help to prevent the downstream effects of ED [35], including both personal and health system burden.

A recent meta-analytic review identified 15 trials that examined whether prevention programs do infact prevent ED onset. There was a significant effect of the prevention programs reviewed, with dissonance-based and lifestyle modification interventions proving most effective, however sensitivity to detecting moderators correlating with larger prevention effects was limited due to sample size, and almost half of these studies had a mean participant age of > 18 at program delivery, past the peak age of ED onset [36].

A systematic review examining cost-effectiveness of ED prevention programs concluded that although the number of published studies on the economic evaluation of ED prevention programs had doubled between 2011 and 2017, they were unable to determine value-for-money of interventions due to heterogeneity of studies [37], highlighting the need for further research in this area. The cost-effectiveness of specific programs is described below.

#### Cognitive dissonance-based programs

Cognitive Dissonance (CD) based programs aim to generate dissonance that reduces an individual’s pursuit of an unattainable and unrealistic thin-ideal, as individuals align their attitudes with their behaviours [10, 38]. Six CD programs were identified within this RR, one example being the *Body Project CD* prevention program, which was developed for a wide range of target groups and which has been extensively delivered online or face-to-face within primary care [39–43]. It comprises four 1-h group sessions involving thought-challenging exercises, open discussion around personal body image concerns, challenging of thin-ideal statements, home exercises and role plays [10].

CD-based programs have the strongest evidence-base of the prevention approaches, resulting in almost 60% reduction in future ED onset in at-risk young females with body dissatisfaction and thin ideal internalisation compared to controls, significantly reducing ED symptoms and risk factors such as dieting, thin idealisation, body dissatisfaction, and negative affect [10, 34, 44]. Dissonance-based interventions have been found to be particularly effective for selective prevention, while Cognitive-Behavioural Therapy (CBT) is more effective for indicated prevention [45]. Successful programs aimed at reducing ED risk factors are often grounded in a cognitive-behavioural theory that contain content on healthy eating and nutrition, media literacy and sociocultural aspects associated with beauty ideals, and body acceptance and body satisfaction [16]; however, both online dissonance-based and online cognitive-behavioural interventions have been found to be effective in lowering ED risk factors in a high-risk population [46] and both approaches and theories should be considered for ED prevention.CD-based preventative approaches have also been shown to be cost-effective. A 2012 cost-effectiveness analysis of the program found that the intervention cost approximately US$70 (A$104) per person to deliver, well below the ‘willingness to pay’ threshold for EDs [39]. Cost-effectiveness modelling of a national school-based CD prevention program in Australia found delivering the program would result in a mean of 16 DALYs (Disability-Adjusted Life Years) averted and would be 80% cost-effective if run for at least five years [47].

A novel dissonance-based intervention with university-aged couples involving interactive role plays, open discussion and interpersonal skills-building significantly reduced numerous key risk factors for EDs including environmental pressures to be thin, internalisation of the thin and athletic ideals, state body dissatisfaction, and actual-ideal body discrepancy [48]. Other smaller trials adapting this content to provide interactive interventions have also demonstrated CD’s effectiveness at reducing ED symptomatology [49]. When delivered by trained facilitators to high school-aged females (i.e., ages 14–18 years), compared with the provision of education brochures, the ‘*Body Project’* CD intervention was found to reduce body dissatisfaction in participants at up to three-years follow-up (FU) and decrease ED behaviours among girls considered ‘high-risk’ at one-year FU [41, 42]. The CD intervention was also found to be effective at reducing social and appearance related anxiety and perfectionism among high school-aged females (i.e., ages 14–18 years) [50]. However, delivery of the ‘*Body Project’* to younger female students (i.e., middle school, ages 11–13 years) was unable to produce the same reductions in body dissatisfaction and ED risk factors [51]. Further analysis and comparison of data from three ‘*Body Project’* trials confirmed the intervention had greater measured effectiveness when delivered to older students (14 years and over) and to those with elevated thin-ideal internalisation at baseline [52]. Evidence suggests that while peak ED onset does not generally occur until late adolescence or early adulthood, girls as young as 6 years old can exhibit body dissatisfaction [53, 54] and peak onset of the most severe eating disorder AN is earlier than other EDs [55, 56], suggesting a mismatch between these facts and at least one study reporting prevention efforts to be most effective when delivered to females at age 14 [57].

A retrospective study of the ‘*Body Project’* CD prevention program compared participants (under 18 years of age) who developed an ED versus those who did not at three-year FU. Non-responders (or those who developed an ED despite participating in the prevention program) were found to have higher levels of thin-ideal internalisation, body dissatisfaction, ED symptoms, and negative affect at baseline compared to those who did not go on to develop an ED [58]. The intervention reduced ED symptoms and risk factors but was unable to prevent onset in this group. A more intensive version of the intervention delivered early to the non-responders with higher baseline symptomatology may have prevented risk exacerbation [58].

In a trial delivered to university students in the US, *‘Project Health’*, a CD-based intervention was compared to an existing *‘Healthy Weight’* lifestyle change program, and a control group where participants watched an obesity education video [59]. Results from the study indicated that while the CD *‘Project Health’* intervention was more effective at reducing obesity onset in participants compared with the *‘Healthy Weight’* intervention, incidence of ED in both intervention groups was the same at two-year FU. However, ED incidence was non-significantly lower in both intervention groups (both at 3%) compared to the control group (9%), indicating potential effectiveness as an ED and obesity prevention program [59].

Analysis of the capacity of CD programs to prevent ED onset by type indicates that risk factors targeted by these interventions are more effective for Bulimia Nervosa (BN), Binge Eating Disorder (BED) and Purging subtypes, than for Anorexia Nervosa (AN) [60]. For individuals at risk of developing AN whose low body weight is due to genetic factors rather than intentional pursuit of the thin ideal, impaired interpersonal functioning and depressive/anxiety symptoms have been identified as relevant targets for future prevention programs [60].

While the majority of CD-based programs target adolescent girls and young women, several trials have also adapted CD interventions for males which focus on varied expressions of body dissatisfaction including muscle dysmorphia [61]. This is important given emerging evidence that EDs are also becoming a concern among males, particularly within older age ranges [62]. In this regard, gender biases in the interpretation of ED symptoms in males have been reported, suggesting an probably underdiagnosis of Other Specified Feeding or Eating Disorder (OSFED) and UFED in a group of Australian university students [63]. In a younger school-based sample, no significant differences in psychosocial impairment as a result of engagement in ED behaviours were observed between genders [64]. Males in this cohort were found to experience equivalent psychological distress and negative impacts on their Quality of Life (QoL) as females, stemming from core ED symptomatologies [64]. Peer-led prevention programs may provide additional benefits to both genders due to the relationship between peer comments, body dissatisfaction, and ED pathology observed in a large community sample [65].

##### Peer-led cognitive dissonance-based programs

Peer-led CD interventions have demonstrated efficacy at reducing ED risk factors and have an additional benefit of higher engagement among students [66, 67]. In a comparison of clinician-led versus peer-led CD interventions, a significant reduction in risk factors and ED symptomatology was observed in both intervention groups compared to controls [68]. At one-year FU, the clinician-led intervention group had sustained reductions compared with the peer-led group; however, the peer-led intervention maintained significantly better outcomes than controls [69]. Peer-led studies with high-risk groups (e.g., athletes) and students have demonstrated efficacy of CD and healthy weight interventions. Significant reductions in shape/weight concern, bulimic symptoms, and negative affect were observed in study of athletes, while a separate study of students achieved; reductions in thin-ideal internalisation, body dissatisfaction, dietary restriction, and bulimic symptoms. Gains were maintained to 12 and 14 month FU, respectively [67, 70].

A study comparing the effectiveness of a mixed gender adaptation of the’*Body Project’* CD peer-led intervention among university students found that participation in the program had a greater benefit for males than females [71]. Compared with females where only small reductions in ED symptomatology were observed at post-intervention, improvements among male participants were much larger and maintained to six-month FU [71]. In a peer-led CD intervention for gay males, significant reduction in body dissatisfaction, drive for muscularity, dietary restraint, and bulimic symptoms was observed compared with controls. However, the study only included a four-week FU period, therefore, longer-term outcomes of the interventions are unknown [72]. When the *‘Body Project’* programme was evaluated among high school females in a pilot RCT, the improvements in body dissatisfaction, thin ideal internalization, dietary restraint, and loneliness in the intervention arm suggest that the programme was effective compared to the control arm [73].

A further peer-led CD intervention, *‘REBeL’,* used a module based, self-selection model of prevention delivered in high schools. This differs in format from other programs identified which utilised either a targeted or universal approach [74, 75]. In this program, peer-educators self-selected to provide the intervention. Preliminary results showed decreases in ED risk factors and increases in empowerment indicating potential benefit for this model [74, 75].

##### Online cognitive dissonance-based programs

Results from studies delivering the *‘Body Project’* intervention to university-aged students (i.e., over 18 years) [40, 46, 76–78], including several trials of the intervention delivered online (*‘eBody Project’*), indicate that regardless of whether students enjoy the program, CD can be highly effective at reducing ED symptomatology, including body dissatisfaction and thin-ideal internalisation. This indicates its potential benefit as a prevention program delivered to a wide range of schools and universities [79]. Observed effects of the *‘eBody Project’* intervention also include prevention of weight gain in participants and reduction in ED symptoms [40]. Due to the similar targets and shared characteristics of ED and obesity prevention programs, there is growing literature relating to combining these interventions [80] as they have been found to be effective and cost-effective in preventing disordered weight control behaviours and obesity [81, 82].

In contrast, a study conducted in an Australian university cohort examining the effectiveness of a short online CD prevention program compared to an imagery rescripting intervention found participants who received imagery rescripting intervention to have higher body image acceptance than the CD group [83], supporting the use of online-adapted imagery-based strategies to reduce disordered eating and the risk of developing an ED; however, further exploration of imagery rescripting techniques in preventing disordered eating is required [83].

#### Universal multi-risk factor programs

Universal prevention programs appear to be less effective than selective or indicated approaches in reducing risk factors for EDs [13] and comorbidities including depression [84]. However, universal prevention programs such as multi-risk factor school-based programs have high acceptability and can be beneficial for children and adolescents independent of their risk status [85, 86]. A systematic review of universal prevention programs delivered between 2006 and 2017 found that these interventions were able to reduce important risk factors including body dissatisfaction (i.e., thin-ideal internalisation and media internalisation) but had less impact on later ED development [13].

The current review identified 10 school-based prevention programs. One of these projects, *‘Planet Health’*, utilised the principles of social cognitive theory to promote behaviour change in high-school children and found participation in the program to be associated with reductions in disordered weight control behaviours [87]. Another intervention (*‘PriMa’)* involved presenting preadolescent girls with images and information about AN [88]. Following the intervention, participants reported significantly increased knowledge of AN compared to the control group at three-month FU. However, improvements in self-esteem in the intervention group were only observed post-intervention and not at FU, with no significant differences in eating behaviours between the intervention and control groups [88]. A subsequent FU study of *‘PriMa’* eight years after receiving the intervention found that self-esteem was significantly higher in the intervention group compared with the control group, however no significant differences between groups in terms of ED behaviours were found [89].

A similar program, *‘Torera’* (built on *‘PriMa’),* aims to reduce binge eating behaviours related to BN and BED with messaging about self-esteem, physical activity, managing negative emotions and avoiding the cycle of binge eating and dieting [90]. Interestingly, while the intervention was able to produce a significant increase in self-esteem and decrease in disordered eating behaviours across genders post-intervention, median effect of self-esteem on eating behaviours was only observed in females, which was found to be attributable to a lower baseline body-related self-esteem in females compared with males [90]. There is a need for further research into prevention programs targeting young males, with a large survey of school students in Switzerland indicating that half of male students reported eating concerns or unhealthy eating behaviours [91].

The *‘MaiStep’* program was designed for both young males and females, and compared an ED prevention program delivered by psychologists and trained teachers to a universal prevention program for stress prevention delivered by untrained teachers (active control) [88]. Content of the MaiStep program focused on mindfulness, body image, negative emotions, and interpersonal conflict, and included group discussion. Participants who did not display ED symptoms experienced a reduction in body image related thoughts and increase in interoceptive awareness at 12-month FU. However, the intervention was unable to produce any statistically significant improvements among individuals with existing ED symptomatology [92].

Several other teacher-delivered universal programs were found to be beneficial for participating students. A study in the UK delivering a body image-based program to adolescent girls, significantly reduced thin-ideal internalisation; however, no differences were observed in relation to eating pathology when compared to the control group [93]. On the other hand, the *‘POPS-Program’* delivered in the Netherlands, instead of focusing on providing education on ED risk factors or symptoms, focused on self-esteem, body image, and nutrition and resulted in significant reductions on almost all measures of ED symptomatology, maintained at one-year FU [94]. Further investigation of a multi-risk factor body image dissatisfaction prevention program in Australian classrooms found that *‘Happy Being Me’* was effective at increasing self-esteem and reducing thin ideal internalisation, body dissatisfaction, and dietary restraint when delivered as a selective prevention to girls aged 11–14 [95] and as a universal prevention delivered to co-educational groups [96].

Building on a growing evidence base relating to the utility of combining ED and obesity prevention programs for delivery in schools, three studies were identified by this RR, examining the potential effectiveness of these interventions. Two of these studies reported on comparison of three interventions in Australian students [97, 98]. The media-literacy program ‘*Media Smart’* was compared to ‘*Life Smart’* and ‘*HELPP’*, all delivered with the same frequency and duration of lessons (all programs described further in the section below) [97]. Both *Media Smart* and *HELPP* had a focus on reducing ED symptoms, while *Life Smart* had broader ED and obesity risk factor targets. Of the three interventions, *Media Smart* was the only program where both ED and obesity risk factors were reduced among participants [97]. Further analysis of study outcomes found that, among participants with higher shape/weight concern at baseline, participation in *Life Smart* and *HELPP* increased eating concern and led to higher rates of meal skipping compared to controls [98].

An online prevention program delivered at two high schools in the US—*‘Staying Fit’*—offered two separate pathways for universal and indicated delivery based on students’ Body Mass Index (BMI) and appeared to be the only school-based program aimed at reducing unhealthy behaviours associated with BED [99]. *Staying Fit* demonstrates the feasibility of delivering a prevention program with two streams based on assessed risk [99].

#### Media literacy-based prevention

Six articles identified by the RR investigated the effectiveness of media-literacy interventions, with a large proportion of these relating to the *‘Media Smart’* program delivered to adolescents and young adults in Australia and New Zealand [100]. Similar to CD interventions, media-literacy interventions target thin idealisation but adopt a divergent approach, focusing on critical analysis of media content to educate and empower individuals to identify, analyse, and challenge unhealthy and stereotypical messages portrayed in the media [101]. A systematic review found media literacy to be the most effective universal prevention approach, showing significant modest effects on risk factors, and demonstrated positive effects for both males and females, resulting in a reduction of weight/shape concern and media internalisation compared to controls at up to 30-month FU [34, 45].

‘*Media Smart’*, was evaluated with the aim of reducing risk among Australian school children and was found to produce favourable results including reduction in shape/weight concern and a significant reduction in weight-related peer teasing, a known risk factor in the development of EDs [102, 103]. An online adaptation, *‘Media Smart-Targeted’*, when delivered to young adult women with elevated baseline ED risk or already reporting disordered eating behaviours (i.e., a more indicated prevention approach), reduced ED development by 66% within 12 months of intervention compared to controls [100]. A larger RCT comparing ‘*Media Smart-Targeted’* to *‘Student Bodies’* (an online prevention program targeting young adult women seeking to improve their body image) found ‘*Media Smart-Targeted’* to be significantly more effective at reducing ED symptomatology than the comparison intervention [104]. Further, participation in the ‘*Media Smart-Targeted’* program was found to reduce depression at up to 12-month FU [105].

Programs have also demonstrated effectiveness for younger cohorts. A universal prevention program using a media literacy approach delivered to school children (i.e., ages 12–15 years) in Spain found the intervention resulted in significant improvements to beauty ideal internalisation, disordered eating attitudes and weight-related teasing [106] and self-esteem [107] compared with controls over a 12- and 13-month FU period, respectively. Further assessment of participants at 30-month post intervention found that children in the media literacy programme had greater body satisfaction than those in the control group [108, 109]. In a study comparing prevention programs using a media literacy approach (two prevention programmes with and without nutritional education), resulted in reductions in perceived pressure to be thin and improvements in nutritional knowledge, which were consistent across both intervention conditions; however, larger reductions were observed among girls at higher ED risk [110]. More pronounced improvements were also observed among participants with greater engagement in interactive activities involved in the prevention program [111]. A pilot study examining a social media literacy intervention among adolescent females found that those receiving the intervention showed improvements in body image (body esteem-weight), disordered eating (dietary restraint) and media literacy (realism scepticism) compared to the control group [112].

Assessment of the impact of the gender mix of groups on the effectiveness of media-literacy programs indicated that girls who participated in the mixed-gender version of the intervention derived more significant benefit than the girls-only group on almost all measures of media literacy and body image used by the study, potentially due to the positive interactions between genders from typically higher levels of confidence and self-esteem displayed by males [113]. Further research is required to confirm this finding and to identify further aspects of media literacy-based programs for universal prevention optimisation, although extant research in this area shows promise.

#### Mindfulness-based prevention programs

Mindfulness is the practice of focusing one's attention in a non-judgmental way on the present moment, while acknowledging and accepting one's thoughts, feelings, and bodily sensations [114]. Examples of mindfulness training include breathing exercises, meditation, progressive relaxation, autogenic training, hypnosis, imagery, and tai chi [115]. Three studies examining the effectiveness of mindfulness-based prevention compared with other established approaches were identified by the RR. Mixed results were reported by these studies on the benefits of mindfulness to reduce ED and other mental health symptomatology.

A systematic review and meta-analysis of mindfulness-based prevention programmes offers a critique on CD interventions commenting that, while they have demonstrated efficacy among females in late adolescence and early adulthood, there is less evidence of their effectiveness in other demographic groups [115]. Mindfulness interventions were effective in reducing body image concern and negative affect (emotional distress or negative emotions) and increasing body appreciation in female participants compared to waitlist or assessment only control groups. Additionally, compared to CD preventions, there is evidence for greater effectiveness of mindfulness prevention in increasing self-esteem and reducing negative affect among participants. Findings from this review suggest integration of mindfulness techniques with CD could increase effectiveness of prevention efforts [115], especially for higher risk groups like young women.

Several mindfulness-based prevention programs have been trialled in Australia. Two studies compared mindfulness to CD prevention, one in young adult females and one in female high-school students [116, 117] with conflicting results. In the study involving high-school girls (i.e., ages 14–18), significant reductions in ED symptomatology and risk factors were observed across both intervention types compared with control groups [116]. However, in the pilot study involving young adult females (ages 17–31), the mindfulness intervention was significantly superior at reducing ED symptomatology and associated psychological impairment post-intervention compared to the CD intervention where no significant differences in outcomes were noted between the intervention and control groups [117]. In contrast, a mindfulness-based prevention program delivered in an Australian high school found no benefits for participants receiving the intervention compared with controls [118]. Additionally, some individuals who received the intervention reported higher anxiety than those who did not, possibly due to increased awareness of emotional states associated with undertaking mindfulness activities [118].

There is limited evaluation of mindfulness-prevention programs within other settings. In the only study investigating an intervention delivered to women in the workplace, it was found that a 10-week group prevention program based on mindfulness and intuitive eating was able to significantly reduce body dissatisfaction in the intervention group compared with a waitlist control [119]. Although incorporation of yoga and wellness in ED prevention and treatment is being increasingly explored, in a study of a yoga-based wellness and ED prevention program, no significant differences in ED symptomatology were found between the intervention and control groups. However, significant reductions were observed in risk factors including drive for thinness and body dissatisfaction among the intervention group [120]. Similarly, an earlier study of this yoga-based prevention program delivered to a group of ethnically diverse girls found no differences in response to the program based on ethnicity or other socio-demographic factors [121]. Overall, evidence for the effectiveness of mindfulness-based prevention programs, even as an adjunct to other interventions, is limited.

#### Other prevention programs

Prevention programs with novel approaches or conducted in populations not typically targeted by ED programs, or in settings not previously discussed, are summarised below. These studies investigated the effectiveness of programs delivered outside of a school setting; and interventions delivered to younger children and physical education teachers, providing evidence for the potential application of a wide range of approaches to target known ED risk factors. Three studies were categorised to this diverse subgroup within the rapid review.

To test the potential efficacy of a selective prevention program delivered outside of a classroom to both primary- and high-school-aged girls (between ages 10 and 16 years) in Australia, a 10-week intervention, ‘*Girls on the Go!’,* aimed at improving body image was delivered at a local community centre in Melbourne [122]. Participation in the program resulted in significant improvements to self-esteem, indicating the program could be feasibly and effectively delivered outside of a school environment [122].

One of the few programs for early primary school children is the ‘*ABC4YC*’ program. In Australia, it was developed in response to a lack of access to universal prevention programs for primary school aged children (i.e., ages 5–8 years) and was found to significantly improve self-esteem in participants. Program content included education on body diversity, non-appearance related qualities, and non-appearance related functions of the body [123].

Increased risk for ED and excessive exercise has also been observed among physical education teachers in Australia [124]. Delivery of an intervention which combined evidence-based approaches, media-literacy, and CD among this cohort was able to demonstrate the greatest effectiveness at reducing ED symptoms and risk factors [124]. Selective intervention programs have also been shown effective with professional ballet dancers (15 + years later) who reported fewer bulimia-related thoughts and behaviours during and following the intervention [125].

Diabetes is considered a risk factor for the development of ED in adolescents. To address this risk, a pilot program was developed in Australia with the objective of preventing ED onset among girls with type 1 diabetes [126]. The brief interactive intervention which comprised characteristics of previously successful ED risk factor reduction programmes targeting perfectionism, media literacy, and self-esteem in young adolescents, was found to increase self-esteem and self-efficacy related to diabetes management among participants, demonstrating potential benefit as a program for wider delivery to adolescents with diabetes [126].

##### Mode of delivery

Assessment of evidence from online prevention programs indicated that, while they were generally less effective than face-to-face interventions, they provided an essential service as part of a stepped care model for ED [127]. On the other hand, evidence from a meta-analysis of 20 studies found that internet-based prevention programs were effective at reducing ED symptoms and risk factors with small to moderate effect, however, as the evidence base was so small, no firm conclusions can be drawn from such treatment studies [128, 129]. There was more evidence to support internet-based prevention than intervention, particularly for the *‘Student Bodies’* program, which produced a small reduction in ED psychopathology and levels of weight concern and drive for thinness [128–130].

A further internet-based, cognitive behavioural, indicated prevention program was identified for individuals with AN [131]. This intervention included elements of motivational interviewing and content on psychoeducation, media literacy, coping with negative emotions, healthy eating, and exercise with a specific focus on restrictive eating. This intervention was a pilot study with a small sample size and no control group and showed a reduction in dietary restraint and increased BMI in participants, although increased drive for thinness occurred during this weight increase. Further research is required to confirm findings for this intervention [131].

### Early intervention

Evidence from three reviews identified by the RR suggest that early intervention initiatives provided within the first three years of onset of ED symptomatology may reduce delays in help-seeking by: (1) targeting parents and helping them recognise early signs of ED during peak time of onset in adolescence [132]; (2) increasing motivation for change among patients with ED [133]; and (3) addressing stigma and shame associated with ED pathology [134].

Evidence from a systematic review and meta-analysis indicated that some approaches to stigma reduction in ED may be more effective than others, with education about the biological underpinnings of EDs having a small to moderate impact on attitudinal stigma toward EDs [135]. However, the majority of the studies reviewed were conducted in student populations and thus may not be generalisable or produce sustained attitudinal and behavioural change in wider community populations [135]. This review highlights the need for more research to identify more effective approaches to reduce stigma in ED.

#### Early recognition and treatment

An assessment of 140 AN patients found that the mean duration of untreated illness in this group was 25 months with a range of 0 months to 16.2 years, with individuals with early onset (under 14 years old) experiencing the longest mean duration [20]. As most participants in the study had been diagnosed and referred to specialist treatment by their general practitioner (GP) or paediatrician, this study highlights the importance of educating clinicians on EDs and including parents and teachers in prevention and early intervention initiatives. Encouragement of help-seeking behaviour is particularly important considering the study also found that individuals with earlier onset AN are more likely to be responsive to external motivators [20].

A public health intervention *‘Psychnet’* delivered in Germany with the objective of facilitating early recognition and treatment in individuals with AN consisted of several different components delivered over a year. These included a public health literacy campaign; an internet-based treatment guide for individuals with ED, their families, and healthcare professionals; a CD prevention program delivered in schools to adolescents; establishment of multidisciplinary networks of practitioners meeting quarterly to discuss the intervention and present ED cases; and implementation of a specialist AN outpatient service [129]. This intervention was unable to demonstrate any reductions in duration of untreated illness among individuals with AN in the sample population or reductions in the time between disorder onset and first contact with services [136]. However, given the small sample of participants involved in the ‘post-intervention’ group (*n* = 18), findings are difficult to interpret and should be considered in the context of the broader body of literature.

For instance, results from a pilot study of a novel transdiagnostic First Episode and Rapid Early Intervention (FREED) service for EDs, delivered to young adults with an ED duration of ≤ 3 years, had far more promising results in increasing engagement with services [130]. This intervention took a holistic, person-centred approach to provide evidence-based psychotherapy tailored to the individual. Participants in the intervention group had a mean wait time of 42 days between referral and initial assessment compared to 62 days in the control group [137]. Psychological interventions provided to participants with an emerging ED produced significant reductions in ED symptomatology and increases in BMI for individuals with AN, while their carers demonstrated improved general psychopathology, expressed emotion, and less accommodation of ED symptoms [137].

Single-session interventions (SSIs) have been examined as an alternative to costly, time-consuming multi-session treatment protocols. A 2017 meta-analysis reported on 50 RCTs involving over 10,000 youths, finding that SSIs can be effective at reducing psychiatric dysfunction, particularly anxiety, however overall effects were smaller than those observed for multisession treatment protocols [138]. In response to long waiting list times at specialist ED clinics in Western Australia, a single-session psychoeducation intervention was delivered to patients referred to a major ED clinic who were placed on a waitlist for services; this was incorporated into their assessment appointment [139]. Delivery of this single session intervention was found to achieve a reduction in objective binge eating episodes, self-induced vomiting, and overeating in participants, and resulted in a decrease in waitlist time and dropout rates [139].

#### Disordered eating interventions

Two studies were identified investigating use of an Interpersonal Psychotherapy (IPT) to reduce loss of control overeating (LOC-eating) as a risk factor for BED in children. In both the pilot [140] and a parallel-group RCT [141], researchers sought to prevent the development of BED in participants who displayed LOC-eating using two interventions: a family-based interpersonal therapy (FB-IPT) and a health education intervention. In the pilot, reductions in likelihood of LOC-eating, depression, and anxiety were shown in the group of children receiving IPT versus the health education intervention [140], and a reduction in number of objective binge-eating episodes was also observed in the IPT intervention group [134]. Measurement of the reduction in BMI of study participants found that both IPT and health education were effective with no difference between groups [141].

In older age groups, studies suggest that body dissatisfaction and disordered eating continue to persist in midlife as body dissatisfaction is closely associated with perceptions of aging and the accompanying changes in appearance [142]. An early intervention program targeting women in mid-life (i.e., ages 30–60 years) found a significant reduction in body image concern and disordered eating among participants in the intervention group compared to the controls. This was the only early intervention identified by the RR targeting older adults and findings from this study indicate that further research in this population is warranted [142].

In the only study identified by the RR relating to interventions targeted towards culturally and linguistically diverse (CALD) groups, Mazzeo et al. [143] assessed the efficacy of a Dialectical-Based Therapy (DBT) against a weight management program for adolescent girls engaging in binge and LOC eating. Significant improvements were observed in both groups in terms of ED symptoms including weight/shape concern and dietary constraint, as well as negative affect [143]. There is a significant paucity of research of prevention and early intervention in CALD and other diverse groups.

#### Online interventions

A considerable number of early intervention studies identified by the RR were delivered online. Although some of the internet-based early intervention programs discussed were unable to show significant benefit, combined screening and early intervention programs such as ‘*Healthy Body Program’* and ‘*ProYouth’*, present a valuable opportunity for early intervention in large proportions of the population who may not wish to engage in face-to-face services [144]. Low engagement with face-to-face treatment is a common challenge encountered within specialist ED services. *‘MotivATE’* a web-based intervention designed to increase treatment adherence was delivered at an ED service in the UK, to participants prior to their first appointment. Although uptake of the pre-assessment *MotivATE* program was low, individuals who registered for and completed the intervention were almost ten times more likely to attend their assessment appointment than those who did not register [145]. It was suggested that low overall uptake may have been a consequence of researchers choosing not to actively recruit for the intervention or simply being attributed to general low motivation among individuals with EDs to change, but preliminary findings are promising.

Evidence from other online interventions aiming to reduce ED symptoms and increase motivation to change among participants through online engagement reported more promising results. An early study comparing online and face-to-face CBT delivered to Australian women with body concerns and disordered eating, ‘*Set Your Body Free’*, found significant improvements in both groups in body dissatisfaction compared with the delayed treatment control. Post-treatment improvements were greater in the face-to-face than online intervention, however, no significant differences between groups in symptom improvement were evident at six-month FU [146], so long-term benefits are still unclear.

A multi-session online program, *‘ESS-KIMO’*, with therapist feedback for females with symptoms of AN or BN, aimed to help these participants recognise the negative impact of their ED symptoms and increase motivation to change. Following the intervention, women receiving the intervention had lower measured dietary restraint and increased self-esteem and were also more likely to perceive their behaviours as a problem compared with participants in the control group [147, 148]. Like most studies, longer term FU to evaluate ongoing outcomes were not conducted.

In a trial in the Netherlands designed to support individuals in the community with bulimic symptoms (without any formal BN diagnosis), online CBT with therapist support was compared with CBT-based bibliotherapy without therapist support, and a waitlist control condition [149]. A significant reduction in binge/purge frequency as well as ED symptomatology was observed in the online CBT group compared with both bibliotherapy and waitlist control groups. While superiority of the online CBT intervention was reported, lack of therapist support provided to the bibliotherapy comparator suggests this may be the critical point of difference in efficacy between the two interventions rather than the medium through which the intervention was delivered [149].

*‘Parents Act Now’*, a multi-national internet-based early intervention, was conducted across two sites in the US and Germany [150]. The intervention was aimed at parents of female adolescents screened as ‘at-risk’ for AN. The intervention was FBT-based and resulted in favourable outcomes for completers with 35% achieving a reduction in AN risk [150]. However, some parents who were interested in accessing FBT were unable to in their local area, indicating a need for early intervention programs to consider linkages with health system services rather than acting as standalone programs. ‘*E@T’,* another internet based FBT intervention, delivered to girls at risk of AN, resulted in significantly increased weight within the intervention group compared to waitlist controls. However, no significant differences were observed relating to other ED risk factors or symptoms measured including excessive exercise, subjective binge eating, or fasting [151]. In many instances, early prevention programs may suit a suite of interventions to provide options for individual cases but may have limited effect if presented in isolation.

A meta-analysis of data was conducted on eight RCTs delivering the *‘StudentBodies’* internet-based indicated prevention program designed for women displaying ‘subclinical’ ED symptoms [152]. The analysis found the *StudentBodies* program to significantly reduce negative body image and drive for thinness compared with controls at FU (10 weeks to 12 months) with moderate effect sizes [152]. Long-term FU of students participating in ‘*StudentBodies’* at three years post intervention found that 11.2% had developed full or subthreshold BN or BED [153]. Further research assessing the effectiveness of the moderated discussion group feature of the ‘*StudentBodies’* intervention found significantly less weight concern among the group who participated in the guided discussion than those who did not [154]. An expanded form of the ‘*StudentBodies’* intervention, *‘IaM’* for ‘high-risk’ women with comorbid depression, was unable to produce a protective effect over the control group for ED despite significantly reducing weight/shape concern among participants [104]. Assessment of the relationship between weight/shape concern and depression in participants over 24 months found the symptoms to be reciprocal with weight/shape concern predicting onset of depression and anxiety, which then increased weight/shape concern [155].

#### Publicly-targeted interventions

Research has shown that providing information on mental health first aid may increase the confidence of members of the public to assist individuals who are developing a mental illness or experiencing a mental health crisis [7]. Researchers have developed ED specific first-aid guidelines in consultation with clinicians, consumers, and carers [156]. Piloting of a program delivering this ED-specific mental health first aid training to participants resulted in significant increases in problem recognition and knowledge maintained at six-month FU [157]. Approximately 27% of participants also reported providing first-aid to a person with a suspected ED during this FU period, with seven of these individuals progressing to seeking professional help [157]. Additional research reported 85% of participants who underwent ED-specific first-aid training were able to assist others in the three-month FU period [151]. Participants also showed increased knowledge regarding BN and BED, including symptom recognition [158, 159]. Importantly, 91.9% of participants responded that they were more likely to approach someone exhibiting ED behaviours because of attending the three-hour workshop [158]. This work suggests a single session of ED-first-aid training could increase help-seeking among individuals with a suspected ED as a result of the increased capacity of their friends and family members to approach them about their eating behaviours, an important early intervention strategy [157, 158].

Early intervention programmes are often under-evaluated in other ethnic and minority groups, across various age ranges and males, which are important sociodemographic factors that can affect the probability of seeking treatment [159, 160]. Similarly, risk factors, such as eating and feeding difficulties in childhood, should also be considered as they can predict ED symptomatology in adolescence and early adulthood [161] and may require more targeted intervention. Another study also found that those adolescents who did not recognise having disordered eating were less likely to seek mental health treatment [162], which highlights the importance of focusing on health promotion for better outcomes.

## Discussion

This rapid review aimed to provide a broad synthesis of the literature relating to prevention and early intervention initiatives for EDs. There is considerable evidence relating to prevention programs for EDs, particularly around cognitive dissonance-based strategies, universal multi-risk factor prevention programs (such as school-based prevention programs), media-literacy based prevention, mindfulness-based prevention programs and other novel prevention programs conducted in uncommon settings and/or targeting populations not typically targeted by ED programs. The RR also identified several early intervention programs with a considerable number of interventions delivered online, primarily targeting AN and BN. Evidence from these studies suggest that early intervention efforts, particularly when delivered within the first three years of ED onset, may increase motivation and help-seeking behaviour among individuals, reducing DUED.

In the last two decades, eating disorder prevention interventions have advanced considerably, with successful programs, such as cognitive dissonance, cognitive-behavioural therapy and media literacy, demonstrating significant beneficial impacts on ED risk factors and symptom reduction [16, 34]. Most prevention and early intervention programs identified in this RR focused on reducing one or more ED risk factors, such as thin-ideal internalisation, body dissatisfaction, negative affect, dietary restraint, shape/weight concerns and preventing future ED onset. However, it is important to note that many of the studies captured by the review report on the reduction of risk or putative vulnerabilities to ED, and due to the short duration of most studies (ranging from 3 months to 3 years), there is insufficient evidence to demonstrate whether preventing and reducing ED risk factors and symptoms does indeed have an impact on future ED onset.

Several promising universal, selective, and indicative prevention approaches were identified in this review. Notably, there is strong evidence for the effectiveness of cognitive dissonance prevention approaches targeting thin-ideal internalisation and body image concern, which are considered potential predictors for AN, BN, and BED onset [10, 34, 60]. CD programs, designed to reduce subscription to the thin-ideal, were considered to be most effective as universal, selective, and indicated preventions, resulting in significant reduction in ED risk factors, symptoms, and future ED onset [10, 45]. CD programs were found to be highly efficacious among university aged couples, adolescents, and young adult females [38, 48, 163]. Additionally, there is a need to explore the effectiveness of these interventions in broader populations, age groups, and cultural settings. It would be advantageous to study FU periods longer than 3 years (the longest FU identified by the review), to understand the long-term effectiveness of these programs [10].

The target population for most prevention studies was female high school and university-aged students. Younger females and males were rarely targeted, and there is some evidence that programs we do have may not be as effective in these age groups, begging the question as to whether we are developing and testing prevention programs early enough to really prevent the onset of eating disorders which peak in early and later adolescence. Older female adults were also rarely targeted, with only one study exploring the association between aging and increased body image concern and disordered eating in females in midlife [142]. The point prevalence of ED in women in midlife has been estimated at around 4% [164, 165]. Interventions targeting middle-aged females have been shown to be efficacious, leading to clinically significant differences in body image concerns and disordered eating, and emphasising the need for further research in this cohort [142]. Further, EDs in men have become increasingly prevalent in recent decades, with males now representing around 33% of all ED cases [72, 91]. Research suggests that males with ED experience similar levels of detrimental impact on quality of life (QoL) as females with ED, and may yield greater benefits (including symptom reduction) from effective prevention initiatives [64, 71], but may require programs specifically designed for them. Gay men are at particular risk of ED, with demonstrated higher levels of risk compared to heterosexual males [72]. Thus, ED prevention efforts should aim to be inclusive and take a gender and sexuality-sensitive approach. Research is needed to examine the feasibility, acceptability, and efficacy of dissonance-based interventions in such populations.

Evidence has shown the benefits of implementing effective prevention programs that jointly target major public health concerns with shared risk factors, such as obesity, diabetes, and EDs. Individuals with obesity and type 1 diabetes mellitus (T1DM) are at a higher risk of developing EDs, with an increased risk of morbidity and mortality. It is therefore crucial to focus on prevention, early detection, and intervention for this population for lasting health and recovery. Combining ED and obesity prevention programs could be efficacious in reducing ED risk factors, symptoms, and disordered weight control behaviours in people of higher weight [40, 59, 81, 82, 97, 166]. Similarly, for adolescents with T1DM, effective prevention programs can make positive impacts on their self-esteem and self-efficacy related to diabetes management and protective factors for disordered eating [126].

A significant proportion of reviewed interventions took place in schools, universities, within the community, or in outpatient treatment settings. Multi-risk factor universal prevention programs such as school-based interventions were highlighted in this rapid review due to their high acceptability and benefits for both children and adolescents. Although universal prevention interventions are, by design, unable to produce large effect sizes, they can provide long-term benefits when delivered in an interactive, multi-session format by a professional [13, 34]. School-based prevention programs provide an opportunity for wider reach and have been found to significantly increase self-esteem, reduce ED risk factors and symptomatology [90, 93–96, 123]. However, scalability of such programmes is limited, and acceptability, feasibility, and efficacy are varied [93]. Among the interventions, media literacy, when universally delivered, has been found to be an effective intervention for improving ED symptoms, encouraging behaviour change, and reducing risk factors in the long-term leading to sustainable changes in adolescent health [97–100]. While media literacy prevention interventions have been proven effective in improving ED symptoms, further exploration is needed in the context of the new media environment to re-evaluate and refine these interventions to maximise their effects [167].

Several other school-based programs have been evaluated with children and adolescents, which were outside the scope of this review due to eligibility criteria (i.e., from 2009), but are noteworthy of mention. The ‘New Moves’ obesity prevention program was evaluated in a RCT with adolescent females, where active arm participants experienced positive changes in their physical activity, eating patterns and self-image, and their behaviour towards physical activity changed during the intervention, compared to the control arm [168], indicating high feasibility and acceptability among adolescents. Another quasi-experimental multi-component program among elementary school students, ‘Very Important Kids’, demonstrated that students receiving the intervention were less likely to be teased about appearance and weight, compared to the control group, highlighting the importance of implementing prevention programs at a young age [169]. In a student-led health promotion program called ‘Healthy Buddies’, older students (4–7th grades) taught younger students (Kindergarten-3rd grade) about nutrition, physical activity, and healthy body image [170]. Following the program, all students receiving the intervention showed a higher knowledge of healthy lifestyle, older students’ BMI and weight increased less, and younger students’ height increased more in the active arm, compared with control students [170]. These programs showcase the feasibility and efficacy of implementing school-based programs to elementary and middle school students and involving students in peer-taught buddy programs to promote healthy living across a range of school-aged individuals. A significant number of prevention and early intervention studies included in this RR were delivered online. While face-to-face interactions are typically more effective, online programs can provide an accessible, cost-effective service as part of a stepped care model for ED, and may increase treatment engagement among individuals with low motivation to seek help [13, 127, 171]. They may also have a wider reach/increase dissemination, even if less effective than face-to-face delivery.

Limited evidence exists exploring prevention and early intervention programs in other underrepresented populations including CALD populations. This is significant given prevalence rates of EDs vary by ethnicity and race [172]. Further, while most prevention programs target risk factors common to most EDs, there tends to be greater focus on AN and BN, and to a lesser degree BED. Thus, findings may not be applicable across a broader spectrum of ED diagnoses such as ARFID, Purging Disorder (PD), UFED, and OSFED.

While this RR identified several early intervention programs for ED, there is still a paucity of research, with many studies classifying early intervention as within the first three years of full syndrome illness. To develop successful early intervention strategies, further exploration is necessary to better understand the DUED, pathways to care and to identify the perceived barriers to seeking and engaging in evidence-based treatment during the early stages of an ED, including patient-related factors such as lack of recognition of illness severity, low self-awareness, or motivation to seek help and service-level delays while waiting for treatment.

While the effectiveness of delivering interventions designed to encourage early symptom recognition and engagement within at-risk populations has been explored, further research in this area is warranted. Prioritising research into early identification and early intervention may prevent downstream impacts of EDs in this population including decreased motivation for change as the illness progresses. Combining screening and early intervention programs presents a valuable opportunity for early intervention in large proportions of the population who may not wish to engage in face-to-face services [144, 173].

Researchers have indicated a need to replicate and scale-up successful prevention programs as well as identify any potential barriers to wider dissemination to increase their reach [174]. Strategies to increase mental health literacy such as ED-specific mental health training could aid early intervention efforts and encourage help-seeking behaviours and increase engagement with services [11, 175]. First-aid training has been found to increase the knowledge and confidence of individuals in problem recognition and the ability to approach individuals exhibiting ED symptoms and behaviours, encouraging them to seek help [156, 157]. While early intervention can improve prognosis in EDs, long waiting periods for eating disorder services are common and can lead to detrimental impacts on the patient’s health. Evidence suggests that providing early access to intermediary supports or psychoeducation could potentially reduce waitlist time and treatment dropouts.

Capacity to increase the reach of ED prevention programs and enhance treatment uptake are critical for making a significant public health impact and for reducing the burden caused by EDs [176]. While there is a significant knowledge base for effective ED prevention and experience in delivering trials, there remains a lack of translation to clinical and practical settings, highlighting issues around significant time investment required to initiate, and lack of prioritisation in government funding [177]. The need for greater allocation of time and funds to support the implementation of such interventions is crucial to evaluate the long-term efficacy of these interventions.

This RR provides a comprehensive overview of the current landscape of prevention and early intervention initiatives developed and tested over the 13 reviewed years. However, due to the broader scope of the RR, which aimed to inform the national research and translation strategy, this review had several limitations. Broadly defined search terms were used to provide a high-level review of the literature, thus a thorough search using specific or detailed terms was beyond scope. Methodological constraints led to the exclusion of grey literature and unpublished or non-peer reviewed research. Similarly, the RR was limited to English language studies conducted in Western countries, or countries with healthcare systems translatable to an Australian context. This means certain cultural and systemic factors of prevention and early intervention in EDs may have been missed. Nevertheless, this RR has identified important gaps in prevention and early intervention research in EDs and highlighted areas which require further research and validation. Broadly, this review found that most prevention interventions were theory-driven, interactive, targeted one or more ED risk factors, and had broader dissemination potential with online models, and included a wide variation of content suggesting that a range of programs could have a positive impact on ED pathology across a variety of populations.

## Conclusions

Prevention and early intervention programs have been shown to significantly reduce some risk factors, promote early symptom recognition, and encourage help-seeking behaviour for people with EDs, however, existing studies have mostly been conducted in cohorts past the age of peak onset and relatively short FU periods mean there is a lack of information on the long-term impacts of the interventions. Effective development and dissemination of successful prevention and intervention strategies necessitates further research, conducted in younger age groups, into early stages of illness, pathways of care and potential barriers to accessing evidence-based treatment and care, especially within identified high-risk groups.

## Supplementary information

**Additional file 1.** PRISMA flow diagram.

**Additional file 2.** Table of Included studies.

## Data Availability

Not applicable—all citations provided.

## Abbreviations

AN: Anorexia nervosa
ARFID: Avoidant/restrictive food intake disorder
BED: Binge eating disorder
BMI: Body mass index
BN: Bulimia nervosa
CALD: Culturally and linguistically diverse
CBT: Cognitive behavioural therapy
CD: Cognitive dissonance
DALY: Disability-adjusted life year
DBT: Dialectical-based therapy
DSM-5: Diagnostic and statistical manual of mental disorders, 5th edition
DUED: Duration of untreated eating disorder
ED: Eating disorder
FBT: Family based therapy
FB-IPT: Family-based interpersonal therapy
GP: General practitioner
HMA: Healthcare management advisors
IOI: InsideOut Institute for Eating Disorders
LOC: Loss of control
OSFED: Other specified feeding or eating disorder
PD: Purging disorder
QoL: Quality of life
RCT: Randomised controlled trial
RR: Rapid review
TAU: Treatment as usual
UFED: Unspecified feeding or eating disorder
