# Supplementary material for: Prevention and early intervention in eating disorders: findings from a rapid review
Source: J Eat Disord. 2023 Mar 10;11:38. doi: 10.1186/s40337-023-00758-3 (PMC9999654; doi:10.1186/s40337-023-00758-3)
Supplement: Supplementary file 1 — Additional file 1. PRISMA flow diagram. [file 40337_2023_758_MOESM1_ESM.docx]

Additional File 1. PRISMA flow diagram

Articles identified through database searching

(n=17,757)

Articles identified through links and reference lists

(n=36)

Articles after duplicates removed

(n=9,260)

Articles screened through assessment of abstract/title

(n=9,260)

Excluded

(n=7,292)

Full text articles assessed for eligibility

(n=1,968)

Excluded

(n=660)

Expert research collaborative requested articles

(n=19)

Articles included in Rapid Evidence Review

(n=1,327)

Identification

Screening

Eligibility

Included
