# Supplementary material for: Prevention and early intervention in eating disorders: findings from a rapid review
Source: J Eat Disord. 2023 Mar 10;11:38. doi: 10.1186/s40337-023-00758-3 (PMC9999654; doi:10.1186/s40337-023-00758-3)
Supplement: Supplementary file 2 — Additional file 2. Table of Included studies. [file 40337_2023_758_MOESM2_ESM.docx]

**Additional File 2. Tables of Included studies**

**Table 1:** Studies included in the rapid review categorised by prevention programs

| **Author** | **Country** | **N participants** | **N studies** | **Population** | **Aim** | | **Design** | **Outcome measure** | |
| --- | --- | --- | --- | --- | --- | --- | --- | --- | --- |
| **Prevention (*n*=96)** | | | | | | | | | |
| *Cognitive Dissonance-Based Programs (n=31)* | | | | | | | | | |
| Akers et al., 2017 [39] | USA | 408 |  | Community (Adult, Females) | To report the cost-effectiveness of the Body Project for clinically meaningful subthreshold symptoms reduction using data from a trial evaluating its effectiveness when delivered by college clinicians | | Modelling (Statistical) | Cost effectiveness (cost per participant, incremental cost, effectiveness: % achieving change*, incremental effectiveness, incremental cost per individual CMC) | |
| Becker et al., 2012 [67] | USA | 157 |  | Community (Adult, Females) | To investigate whether two evidence-based programs appear promising for future study if modified to address the unique needs of female athletes | | Longitudinal (<5yr) (Intervention: 3 weeks; Follow-up: 1-year) | Thin ideal internalisation, dietary restraint, bulimic pathology, body dissatisfaction, negative affect, manipulation check | |
| Bentley et al., 2015 [64] | Australia | 1666 |  | Community (Adolescents, Both sexes) | To examine sex differences in psychosocial impairment associated with eating disorder features (EDF) in adolescents. | | Cross-Sectional | EDF (extreme dietary restriction, objective binge eating, subjective binge eating, purging, excessive exercise, and weight/ shape overvaluation) and psychosocial impairment (general psychological dis- tress and quality of life) | |
| Brown & Keel, 2015 [72] | USA | 87 |  | Community (Adult, Males) | To investigate the acceptability and efficacy of a cognitive dissonance-based (DB) intervention (The PRIDE Body Project) in reducing eating disorder risk factors among gay males in a university-based setting | | RCT (Intervention: 1 week; Follow-up: 1 month) (Incentive: yes) | Body dissatisfaction, drive for muscularity, body-ideal internalisation, dietary restraint, bulimic symptoms, self, and partner objectification | |
| Brown et al., 2017 [61] | USA | 52 |  | Community (Adult, Males) | To investigate the acceptability and efficacy of a cognitive dissonance-based (DB) intervention in reducing eating disorder and muscle dysmorphia risk factors in men with body dissatisfaction. | | RCT (Intervention: 1 week; Follow-up: 1 month) (Incentive: yes) | Body internalisation, dietary restraint, bulimic symptoms, muscle dysmorphia symptoms and drive for muscularity | |
| Christian et al., 2019 [50] | USA | 332 |  | Community (Adolescents, Females) | To examine ED symptoms, comorbidities, and transdiagnostic risk pre- and post-Body project and at 1 month follow up | | RCT (Follow-up: 1 month) (Incentive: yes) | ED symptoms, drive for thinness, body dissatisfaction and bulimic symptoms. Thin-ideal internalisation, exercise dependence, eating disorder comorbidities, appearance anxiety, worry, rumination, depression, eating disorder risk and transdiagnostic factors, shame, and guilt | |
| Dakanalis et al., 2019 [10] | Switzerland |  | N/A | Community (Young Adult, Females) | To briefly document progress in UK, Canada and Australia using the dissonance-based ED prevention approach | | Review (Narrative) | ED symptom reduction, thin-ideal internalisation, body dissatisfaction | |
| Dion et al., 2016 [54] | Canada | 1515 |  | Community (Children, Both sexes) | To assess body dissatisfaction among children between 9 and 14 years of age and to examine factors (age, sex, body mass index, perceived shape, and self-esteem) associated with wanting a thinner or a larger shape | | Cross-sectional | Sociodemographic variables, anthropometry measurements, Culture Free Self-Esteem Inventories-2, Contour Drawing Rating Scale, | |
| Favaro et al., 2009 [55] | Italy | 1666 AN; 793 BN |  | Community (Mixed cohort, Both sexes) | To explore the time trends in age at onset of anorexia nervosa and bulimia nervosa | | Ecological | Age at onset, socioeconomic status, age at menarche, and number of siblings | |
| Forney, Holland & Keel, 2012 [65] | USA | 2060 |  | Community (Adult, Both sexes) | To examine the influence of peer context on the relationship between body dissatisfaction and eating pathology in females and males. | | Longitudinal (Follow-up: 20 years) | Risk factors and disordered eating levels & influence of peer comments on body dissatisfaction and eating pathology (age, BMI, Body dissatisfaction, friend comments, bulimia, drive for thinness) | |
| Green et al., 2017 [49] | USA | 47 |  | Community (Adult, Females) | To conduct a preliminary randomised controlled trial of a modified dissonance-based eating disorder program vs assessment only condition | | Longitudinal (Intervention: 4 weeks; Follow-up: 2 months) (Incentive: yes) | Body dissatisfaction, self-esteem, self-objectification, thin ideal internalisation, maladaptive social comparison, trait anxiety, eating disorder symptoms | |
| Horney, Stice & Rohde, 2015 [58] | USA | 236 |  | Community (Mixed Cohort, Young females) | To investigate baseline and acute intervention predictors of DSM-5 ED development during a 3 year follow up among Body Project Participants | | Longitudinal (Follow-up: 3 years) | Thin ideal internalisation, body dissatisfaction, dieting negative affect, eating disorder symptom, DSM 5 eating disorders | |
| Le et al., 2017 [34] | Worldwide |  | 112 | Community (Adult, Both sexes) | To systematically review and quantify the effectiveness of Eating Disorder (ED) prevention interventions | | Systematic Review/ Meta-Analysis (combined) | Reduction of ED risk factors or symptoms | |
| Le et al., 2018 [37] | Australia, New Zealand |  | 13 | Community (Mixed Cohort, Both sexes) | To evaluate the cost effectiveness of ED interventions | | Review (Systematic) | Cost-effectiveness of intervention (mostly interventions costs, and health-care utilisation costs). | |
| Linville et al., 2015 [43] | USA | 66 |  | Outpatient (Young Adult, Females) | To conduct a pilot effectiveness trial of a brief dissonance-based eating disorder preventative program, the body project, when implemented at primary care medical clinics | | Quasi-experimental (intervention) | Thin ideal internalisation, pressure to be thin, dieting, body dissatisfaction, eating disorder symptoms and negative affect | |
| Liu et al., 2019 [53] | China | 5734 |  | Community (Children, Both sexes) | To describe body image perception and dissatisfaction and to examine their effects on HRQoL among primary school students in Guangzhou, China | | Cross-sectional | Sociodemographic variables, body image perception, body dissatisfaction, Health-related quality of life (HRQoL), anthropometry measurements | |
| Müller & Stice, 2013 [52] | USA | 977 |  | Community (Mixed Cohort, Young females) | To investigate factors hypothesized to moderate the effects of a dissonance-based eating disorder prevention program, including initial elevations in thin-ideal internalization, body dissatisfaction, eating disorders symptoms, and older participant age | | Quasi-experimental (Intervention: 4 sessions) | Thin ideal internalisation, body dissatisfaction, eating pathology | |
| Ramirez et al., 2012 [48] | USA | 418 |  | Community (Adult, Both sexes) | To investigate a new dissonance-based prevention program that's based on a dual pathway model of ED within context of an individual's romantic relationship | | Quasi-experimental (Intervention: 2 sessions) (Incentive: yes) | Environmental pressures to be thin, internalisation of the thin and athletic ideals, state body dissatisfaction and actual ideal-body discrepancy | |
| Rohde et al., 2014 [51] | USA | Study 1= 81, Study 2= 52 |  | Community (Adolescents, Females) | To pilot a trial that evaluated the efficacy of a dissonance-based ED prevention program for middle school girls with body dissatisfaction | | RCT (Intervention: 6 weeks; Follow-up: 3 months)  (Incentive: yes) | Thin ideal internalisation, perceived pressure to be thin, body dissatisfaction, dieting, negative affect, eating disorder symptoms | |
| Rohde et al., 2015 [57] | USA | 496 |  | Community (Adolescents, Females) | To address the research gap of when risk factors for ED emerge or escalate, or when they begin to predict future ED onset. | | Repeated Measure (with 7 follow-ups annually) (Incentive: yes) | Perceived sociocultural pressure to be thin, thin-ideal internalisation, body dissatisfaction, dieting, negative affectivity, body mass index, eating pathology | |
| Rohde et al., 2018 [59] | USA | 364 |  | Community (Adult, Both sexes) | To test hypothesised mechanisms underlying the effects of two interventions targeting both obesity and eating disorders | | Longitudinal (6 weeks) | Body mass, eating disorder symptoms and diagnosis, cognitive dissonance, physical activity, body dissatisfaction, negative affect | |
| Schoen, Brock & Hannon, 2019 [63] | USA | 237 |  | Community (Adult, Both sexes) | To investigate gender bias, a form of mental health stigma, in perceptions of OSFED/UFED in college students | | Case Study | Gender bias in perception/ratings of vignette characters: eating disorder, food addiction, depression, anxiety, body dissatisfaction, picky eater, weight management, exercise like athlete, healthy. | |
| Seidel et al., 2009 [163] | USA | 71 |  | Community (Mixed Cohort, Young females) | To examine body dissatisfaction as a mediator in partial intervention effects and developed a more effective test of mediation | | Quasi-experimental (Intervention: 4 weeks)  (Incentive: yes) | Treatment and relapse prevention rates, body dissatisfaction, shape concern, dietary restraint, global eating disorder psychopathology, weight concern, purging behaviours i.e., vomiting. | |
| Shaw et al., 2009 [174] | USA |  | N/A | Community (Mixed Cohort, Both sexes) | To analyse eating disorder intervention programs and define success | | Review (Narrative) | Review of ED prevention program | |
| Steinhausen et al., 2015 [56] | Denmark | 5902 AN; 5113 BN |  | Community (Mixed cohort, Both sexes) | To study recent time trends  in the incidence of diagnosed anorexia  nervosa (AN) and bulimia nervosa (BN)  based on nationwide psychiatric register  data. | | Ecological | Age at onset, incidence rate | |
| Stice et al., 2009 [41] | USA | 306 |  | Community (Adolescents, Females) | To investigate the effectiveness of a dissonance-based intervention that is delivered by school staff | | RCT (Intervention: 4 weeks; Follow-up: 1 year)  (Incentive: yes) | Thin ideal internalisation, dieting, body dissatisfaction, depressive symptoms and eating pathology | |
| Stice et al., 2011 [42] | USA | 306 |  | Community (Adolescents, Females) | To test whether this program produces effects through long term follow up when high school clinicians recruit students and deliver the intervention under real world conditions. | | Longitudinal (Intervention: 4 weeks; Follow-up: 3 years)  (Incentive: yes) | Thin ideal internalisation, dieting, body dissatisfaction, depressive symptoms and eating pathology, functional impairment, health care utilisation | |
| Stice et al., 2011 [44] | USA | 306 |  | Community (Adolescents, Females) | To test whether change in thin-ideal internalization partially mediates the effects of the intervention on change in body dissatisfaction and eating disorder symptoms and whether change in body dissatisfaction partially mediates the effects of the intervention on change in symptoms. | RCT (Intervention: 4 weeks; Follow-up: 12 months)  (Incentive: yes) | | Thin-ideal internalisation, body dissatisfaction, ED symptoms |  |
| Stice et al., 2014 [40] | USA | 107 |  | Community (Young Adult, Females) | To evaluate the developed prototype Internet version of a group-dissonance based ED prevention program | | Repeated Measure (with follow-up) (Intervention: 4-6 weeks; Follow-up: 2 years) (Incentive: yes) | Thin-ideal internalisation, body dissatisfaction, dieting, negative affect, ED symptoms | |
| Stice et al., 2021 [36] |  | 5080 | 15 | Community (Young adult, Both sexes) | To provide a review of randomized controlled trials that tested whether an eating disorder prevention program significantly reduced future onset of eating disorders | | Meta-analysis | Future onset of eating disorders | |
| Van Diest et al., 2013 [38] | USA | 177 |  | Community (Adult, Females) | To analyse thin-ideal internalization and self-objectification in a cognitive based eating disorder prevention program and examine how the integration of self-objectification and thin-ideal internalisation predicts body dissatisfaction and eating disorder symptoms | | Longitudinal  (Intervention: 2 sessions; Follow-up: 1 year) | Thin ideal internalisations, body mass index, self-objectification, body dissatisfaction, eating disorder symptoms | |
| *Online Cognitive Dissonance-Based Programs (n=8)* | | | | | | | | | |
| Chithambo & Huey, 2017 [46] | USA | 271 |  | Community (Adult, Females) | To evaluate two web-based programs for eating disorder prevention | | RCT (Follow-up: 4 weeks) | Thin ideal internalisation, body dissatisfaction, dieting, eating pathology, depression | |
| Green et al., 2018 [49] | USA | 82 |  | Community (Adult, Females) | To conduct a preliminary RCT in an expanded online version of the Body Project vs Assessment only control condition in females with clinical and subclinical ED symptoms | | RCT (Follow-up: 2 months) (Incentive: yes) | Self-esteem, body dissatisfaction, social comparison, self -objectification, thin ideal internalisation, ED symptoms, Positive and negative affect schedule, train anxiety, BMI (body mass index) | |
| McMillan, Stice & Rohde, 2011 [77] | USA | 124 |  | Community (Adult, Females) | To evaluate a high-dissonance version of this program against a low-dissonance version and a wait-list control condition to provide an experimental test of the mechanism of intervention effects. | | RCT (Intervention: 4 weeks; Follow-up: 3 months) | Thin ideal internalisation, dieting, negative affect, ED symptoms, body dissatisfaction. | |
| Pennesi and Wade, 2018 [83] | Australia | 107 |  | Community (Adult, Females) | To compare 2 online interventions: imagery rescripting and cognitive dissonances vs an assessment only control | | RCT (< 1 week) (Incentive: yes) | Body image acceptance, negative affect, self-comparison, quality of life, disordered eating | |
| Sanchez-Carracedo et al., 2012 [80] | Spain, USA |  | N/A | Community (Mixed Cohort, Both sexes) | To discuss and review the main reasons for an integrated approach to the spectrum of eating- and weight- related problems, which include anorexia nervosa, bulimia nervosa, anorexic and bulimic behaviours, unhealthy dieting practices, body dissatisfaction, binge-eating disorder, overweight and obesity | | Review (Narrative) | Body mass index | |
| Serdar et al., 2014 [78] | USA | 333 |  | Community (Adult, Females) | To compare the efficacy of an online DB program with a face-to-face DB program and an assessment-only condition | | RCT (Intervention: 3 sessions) | Eating disorder diagnostic scale, ideal body stereotype scale revised, body esteem scale | |
| Stice et al., 2014 [40] | USA | 107 |  | Community (Adult, Females) | To compare the effects of a new internet-based version of a group-based eating disorder prevention program vs control condition educational video and educational brochure) at 1-year and 2-year follow up | | Longitudinal (Follow-up: 2 years) (Incentive: yes) | Thin ideal internalisation, dieting, body dissatisfaction, body mass, depressive affect and eating pathology | |
| Stinson et al., 2018 [79] | USA | 170 |  | Community (Adult, Females) | To investigate whether credibility, expectancy, and acceptability of the Body Project is impacted by level of disordered eating pathology and whether perceived credibility, expectancy, and acceptability impacts treatment outcomes. | | Repeated Measure (with follow-up) (Intervention: 2 sessions; Follow-up: 4 months) (Incentive: yes) | Credibility, expectancy, acceptability, thin ideal, body satisfaction, restraint, weight concerns, shape concerns, eating concerns, routine restraint, compensatory restraint, external cues, emotional eating, BICS | |
| *Peer-Led Cognitive Dissonance-Based Program (n=16)* | | | | | | | | | |
| Agam-Bitton, Ahmad & Golan, 2018 [113] | Israel | 259 |  | Community (Adolescents, Females) | To examine the preferred setting for a school-based wellness programme called “In Favour of Myself" | | RCT (Intervention: 9 weeks; Follow-up: 3 months) | Self-esteem, media literacy, sociocultural attitudes towards appearance, drive for thinness and body dissatisfaction, body esteem | |
| Becker et al., 2010 [70] | USA | 106 |  | Community (Adult, Females) | To modify Healthy Weight (MHW) eating disorder prevention to facilitate peer delivery, elaborating on benefits of healthy ideals and consumption of nutrient dense food. | | Longitudinal (<5yr) (Intervention: 2 sessions; Follow-up: 14 months) | Negative affect, thin ideal internalisation, body dissatisfaction, dietary restraint, bulimic pathology | |
| Breithaupt et al., 2017 [74] | USA | 83 |  | Community (Adolescents, Females) | To determine if an enhanced empowerment model of REbeL could increase feelings of empowerment and reduce eating disorder risk, and to assess the feasibility and acceptability of the intervention | | Quasi-experimental (Intervention: 8 months) | Body esteem, empowerment scale, body surveillance, internalisation of thin ideal, perceived pressures to be thin | |
| Breithaupt et al., 2019 [75] | USA | 82 |  | Community (Adolescents, Females) | To determine whether an after-school ED prevention model, emphasizing empowerment and youth leadership, could be implemented in a high school setting in a self-sustaining fashion and reduce ED risk | | Quasi-experimental (Intervention: 8 months) | Acceptability, empowerment & body esteem | |
| Butryn et al., 2014 [69] | USA | 408 |  | Community (Adult, Females) | To examine facilitator characteristics and group-level variables as potential outcome predictors of an effectiveness trial under more real-world conditions | | RCT (Follow-up: 1 year) | Eating disorder symptoms, thin ideal internalisation, body dissatisfaction, negative affect, BMI | |
| Espinoza, Penelo & Raich, 2013 [108] | Spain | 443 |  | Community (Adolescents, Both sexes) | To assess changes in the body image of Spanish adolescents who participated in a programme aimed at preventing disordered eating, with a 30-month follow-up. | | Repeated Measure (follow-up: 30 months) | Body problems, body satisfaction | |
| Greif, Becker & Hilderbrandt, 2015 [66] | USA | 64 |  | Community (Adult, Females) | To examine the effectiveness of a Train-the-trainer model at reducing risk factors in Body Project participants | | Repeated Measure (Follow-up: 5 months) | Body image dissatisfaction, negative affect, thin ideal internalisation, bulimic pathology | |
| Kilpela et al., 2016 [71] | USA | 115 |  | Community (Adult, Both sexes) | To explore the efficacy of a mixed-gender Body Project compared with the historically female-only body image intervention program. | | RCT (Intervention: 2 sessions; Follow-up: 6 months) (Incentive: yes) | Negative affect, appearance ideal internalisation, body satisfaction, eating disorder pathology, male body attitudes. (demographics and peer leader adherence was also measured. | |
| Lopez-Guimera et al., 2011 [111] | Spain | 263 |  | Community (Adolescents, Females) | To assess impact of a school-based program aimed at preventing disordered eating | | RCT (Intervention: 4 sessions; Follow-up: 6 months) | BMI, EAT, Indicator of adherence to activities (ACT) | |
| Mora et al., 2014 [107] | Spain | 200 |  | Community (Adolescents, Both sexes) | To evaluate the long-term effects of two school-based prevention programs administered to a universal mixed-sex sample of school-going adolescents on disturbed eating attitudes, aesthetic ideal internalization, and other eating disorder risk factors, when compared to a control group. | | Longitudinal (<5yr) (Intervention: 10 weeks; Follow-up: 13 months) | EAT-26: eating attitudes test, SCOFF Questionnaire, Sociocultural attitudes towards Appearance Questionnaire, contour drawing rating scale (CDRS), Rosenberg self-esteem scale | |
| Stice et al., 2013 [68] | USA | 171 |  | Community (Adult, Females) | To test whether undergraduate peer leaders can deliver a dissonance-based ED prevention program which could facilitate the broad dissemination of this efficacious intervention | | RCT (Intervention: 4 weeks; Follow-up: 1 year) (Incentive: yes) | Thin ideal internalisation, body dissatisfaction dieting, negative affect, eating disorder symptoms | |
| Vanderkruik, Gist & Dimidjian, 2020 [73] | USA | 105 |  | Community (Adolescents, Females) | To evaluate using mixed-methods the Body Project program when peer-delivered among high school females | | Quasi-experimental (Intervention: 4 weeks) (Incentive: Yes) | Eating disorder risk factors, social, and self-constructs | |
| Wade et al., 2017 [101] | Australia | 616 |  | Community (Adolescents, Both sexes) | To examine whether media internalization, found to mediate the relationship between selected prevention programs and outcomes, mediated the impact of two universal prevention programs that targeted risk factors for eating disorders and obesity, namely weight concern, and shape concern. | | Longitudinal (>5yr) (Intervention: 8 lessons; Follow-up: 12 months) | Shape concern, weight concern, media internalisation | |
| Wilksch et al., 2015 [97] | Australia | 1316 |  | Community (Adolescents, Both sexes) | To investigate the efficacy of an obesity-prevention program (Life Smart) and two eating disorder-prevention programs (Media Smart and HELPP) against each other and a no-intervention control condition with young adolescent girls and boys from pre- to post-intervention and over a 12-month follow-up. | | RCT (Intervention: up to 34 classes; Follow-up: 12 months) | Weight concern, Shape concern, Eating concern, Dieting, Body dissatisfaction, Media internalization, Perceived pressure, Depression Weight-related peer teasing, Perfectionism, Regular eating, Screen time, Physical activity | |
| Wilksch et al., 2018 [100] | Australia, New Zealand | 194 |  | Community (Young Adult, Females) | To assess the real-world effectiveness of two online ED prevention programs | | RCT (Intervention: 9 modules; Follow-up: 12 months) (Incentive: yes) | Global EDE-Q, secondary: weight concerns, depression, media internalisation, ineffectiveness, clinical impairment, mental quality of life, risk-suicide, drug, alcohol | |
| Wilksch, 2015 [102] | Australia | 51 |  | Community (Adolescents, Both sexes) | To test delivery of Media Smart, school-based ED program, by teachers that's shown success when delivered by professionals | | RCT (Intervention: 2 weeks; Follow-up: 6 months) | Shape and weight concern, dietary restraint, body dissatisfaction, media internalisation, self-esteem, feelings of ineffectiveness, weight related peer teasing | |
| *Media-Literacy Based Prevention (n=8)* | | | | | | | | | |
| Gonzalez et al., 2011 [109] | Spain | 443 |  | Community (Adolescents, Both sexes) | To evaluate the long‐term effects of a school‐based prevention programme administered to adolescents | | Repeated Measure (Intervention: 4 sessions; Follow-up: 30 months) | Eating Attitudes Test, influences of aesthetic body ideal, BMI | |
| McLean et al., 2017 [112] | Australia | 64 |  | Community (Adolescents, Females) | To investigate the effectiveness of a social media literacy intervention for adolescent girls on risk factors for eating disorders. | | Quasi-experimental (Intervention: 3 sessions) | Body esteem, fear of fat, dietary restraint, overevaluation of weight/ shape, internalisation, appearance conversations, upwards appearance comparison, fear of negative appearance evaluation, realism scepticism, critical thinking about appearance media | |
| Raich, Portell & Pelaez-Fernandez, 2010 [110] | Spain | 349 |  | Community (Adolescents, Females) | To evaluate the effectiveness of universal school-based ED prevention administered to female secondary school students | | Quasi-experimental (intervention) (Follow-up: post-intervention) | Eating Attitudes Test, influences of aesthetic beauty model, nutrition, BMI | |
| Sanchez-Carracedo et al., 2016 [106] | Spain | 565 |  | Community (Adolescents, Females) | To assess the effectiveness of a universal ED prevention program for young females delivered by community providers previously trained in an  integrated approach to the prevention of eating and weight-related  disorders (MABIC project) | | Quasi-experimental (Intervention: 8 weeks; Follow-up: 1 year) | BMI, Beauty ideal, Eating attitudes and behaviours, Perception of teasing, Satisfaction with body, Drive for thinness, Possible cases of EDs, Negative Affect, Self-esteem | |
| Wade & Wilksch, 2018 [127] | Australia |  | N/A | Community (Young Adult, Females) | To examine the efficacy of internet prevention in eating disorders that have emerged since 2016 | | Review (Narrative) | Efficacy of Internet prevention in EDs, body image concerns, media literacy, cognitive dissonance, CBT | |
| Watson et al., 2016 [45] | Worldwide | 3989 | 107 | Community (Mixed Cohort, Both sexes) | To evaluate the efficacy of universal, selective, and indicated eating disorder prevention | | Systematic Review/ Meta-Analysis (combined) | Media literacy, dissonance-based ED prevention, CBT, healthy weight program, media literacy, psychoeducation ED preventions | |
| Wilksch & Wade, 2009 [103] | Australia | 540 |  | Community (Adolescents, Both sexes ) | To evaluate a theoretically informed media literacy program delivered to a mixed-sex, universal, young adolescent audience. | | RCT (Intervention: 8 weeks; Follow-up: 30 months) | Shape and weight concern (EDE-Q), dieting, body dissatisfaction (EDI), media internalization, perceived pressure, ineffectiveness, depression, self-esteem | |
| Wilksch, O'Shea & Wade, 2019 [105] | Australia, New Zealand | 316 |  | Community (Adult, Female) | To outline the impact of an online eating disorder risk reduction program on brief, self-report measures of depressive symptoms, alcohol and other drug use, and suicidality. | | RCT (Intervention: 9 weeks; Follow-up: 12 months) | Depression, alcohol use, alcohol dependence, drug use, suicidal thoughts | |
| *Mindfulness-Based Prevention (n=7)* | | | | | | | | | |
| Atkinson & Wade, 2016 [117] | Australia | 44 |  | Community (Young Adult, Female) | To assess the feasibility of a pilot mindfulness-based intervention with respect to reducing the risk of eating disorders in young females. | | RCT (Intervention: 3 weeks; Follow-up: 6 months) | Primary outcomes: Weight and shape concern, negative affect; Secondary outcomes: Dietary restraint, Thin ideal internalization, Sociocultural pressures, Eating disorder symptoms, Psychosocial impairment | |
| Atkinson & Wade, 2016 [116] | England | 347 |  | Community (Adolescents, Females) | To assess the feasibility, acceptability, and efficacy of a novel mindfulness-based intervention for reducing the risk of eating disorders among adolescent females, under both optimal (trained facilitator) and task- shifted (non-expert facilitator) conditions. | | RCT (Intervention: 3 weeks; Follow-up: 6 months) | Weight shape concern, negative affect, dietary restraint, thin ideal internalisation and sociocultural pressures, psychosocial impairment, eating disorder symptoms | |
| Beccia et al., 2018 [115] | USA | 2173 | 20 | Community (Mixed Cohort, Young females) | To evaluate the efficacy of mindfulness-based eating disorder prevention programs | | Systematic Review/ Meta-Analysis (combined) | Body image/dissatisfaction, weight shape concerns, disordered eating drive for thinness, dietary restraint | |
| Bush et al., 2014 [119] | USA | 124 |  | Community (Adult, Females) | To examine the efficacy of a novel intervention for problematic eating behaviours and body dissatisfaction. | | Quasi-experimental (intervention) | Cognitive and behavioural: intuitive eating, body appreciation, mindfulness, eating behaviours | |
| Cook-Cottone et al., 2017 [120] | United States | 170 |  | Community (Children, Females) | To investigate outcomes of a revised version of a yoga-based, eating disorder prevention program, targeting eating disorder risk factors, among fifth grade girls | | Quasi-experimental (intervention) (Follow-up: 10 weeks) | BMI, SES, Eating Disorder inventory-3, Self-care Scale | |
| Cook-Cottone, Jones & Haulgi, 2010 [121] | United States | 50 |  | Community (Children, Females) | To examine ethnic differences in primary prevention programs for eating disorders in young girls. | | Repeated Measure (without follow-up) (Intervention: 10 weeks) | Drive for thinness, bulimia, body dissatisfaction, perceived stress, competence, physical self-concept, social self-concept | |
| Johnson et al., 2016 [118] | Australia | 132 |  | Community (Young Adult, Both sexes) | To assess whether the promising effects of mindfulness-based interventions in schools could be replicated in a randomised controlled trial independent of program developers in an Australian context, and to investigate a broad range of primary outcome measures, including anxiety, depression, wellbeing, and a risk factor for eating disorders (weight and shape concerns), to assess the potential of this intervention as a transdiagnostic prevention program | | RCT (Intervention: 8 lessons; Follow-up: 3 months) | Primary: Depression, anxiety, weight/shape concerns, wellbeing; Secondary: emotional dysregulation, self-compassion, mindfulness | |
| *Other Prevention Programs (n=9)* | | | | | | | | | |
| Bar, Cassin & Dionne, 2017 [125] | Canada | 116 |  | Community (Adult, Females) | To examine whether prevention programs can effectively lower the lifetime prevalence of ED symptomatology amongst elite athletes | | Longitudinal (Follow-up: 15 or more years) | Lifetime prevalence of vomiting and laxative use to control weight | |
| Damiano et al., 2018 [123] | Australia | 51 |  | Community (Children, Both sexes) | To assess whether ABC-4-YC was likely to have a positive impact on young children’s body image attitudes, to assess the feasibility of ABC-4-YC from the point of view of practicality and acceptability to teachers delivering the program in the classroom, and to assess teacher feedback about ABC-4- YC to determine the practicality and acceptability of classroom delivery. | | Quasi-experimental (Intervention & Follow-up: 1 week) | Children’s body dissatisfaction, weight stigma, internalization of appearance ideals, and appearance-based teasing. | |
| Ohlmer, Jacobi & Taylor, 2013 [131] | Germany | 36 |  | Community (Adult, Females) | To develop a prevention program specific for at risk for anorexia nervosa (AN) or with restrictive eating and normal body weight risk groups and to assess its feasibility and effectiveness in a pilot study. | | Quasi-experimental (Intervention & Follow-up: 10 weeks) | BMI; Weight Concerns Scale; EDE-Q Restraint; Beck Depression Inventory | |
| Loucas et al., 2014 [129] | United Kingdom |  | 20 | Community (Mixed Cohort, Both sexes) | To examine effectiveness of randomised control trials (RCT) with e-therapy CBT intervention programs as treatment and relapse prevention | | Systematic Review/ Meta-Analysis (combined) | Efficacy of prevention and treatment ED program | |
| Melioli et al., 2016 [128] | France |  | 20 | Community (Mixed Cohort, Young females) | To evaluate the efficacy of Internet-based programs in decreasing eating disorder (ED) symptoms, and second, to identify moderator variables these effects. | | Meta-Analysis | Body dissatisfaction, drive for thinness and internalisation of the thin ideal; Shape and Weight concerns; Bulimic symptoms, negative affect, and restriction; Purging frequency | |
| Moessner et al., 2017 [176] | Germany | N/A |  | N/A | To create a mathematical model of how healthcare for eating disorders affects the population's disease burden | | Modelling (Statistical) | Incidence rate, spontaneous remission, prevention effect and reach rates, treatment effect, effectiveness, relapse and reach rates, relapse prevention effect and reach rates. | |
| Tirlea, Truby & Haines, 2016 [122] | Australia | 122 |  | Community (Mixed Cohort, Young females) | To test the effectiveness of an intervention delivered by health professionals outside the school environment to girls identified with issues such as poor body image, low self-esteem, low self-confidence, nonparticipation in sports, or being overweight or underweight. | | RCT (Intervention: 10 weeks; Follow-up: 6 months) | Self-esteem, physical health, self-efficacy, mental health self-efficacy, Body image satisfaction, Dutch eating behaviour questionnaire for children (DHEB), clinical impairment assessment (CIA) | |
| Völker, Jacobi & Taylor, 2011 [130] | USA | 22 |  | Community (Adult, Females) | To test the feasibility of the adapted Internet-based prevention program “Student BodiesTM” for females with subclinical ED and to obtain effect size and sample size estimates for a subsequent randomized controlled trial. | | Quasi-experimental (Intervention & Follow-up: 8 weeks) | EDI-2: Eating Disorder Inventory 2; EDE-Q: Eating Disorder Examination Questionnaire; WCS: Weight Concerns Scale; SCL-90-R (GSI): Symptom Checklist Revised (Global Severity Index); BDI: Beck Depression Inventory; BMI: body mass index. | |
| Yager and O'Dea, 2010 [124] | Australia | 170 |  | Community (Adult, Both sexes) | To examine the impact of two interventions on body image, eating disorder risk and excessive exercise among trainee health education and physical education (HE&PE) teachers who were considered an ‘at-risk’ population for poor body image and eating disorders. | | RCT (Intervention: 2 weeks; Follow-up: 6 months) | Self-esteem, disordered eating, and excessive exercise behaviours | |
| *Universal Multi-Risk Factor Programs (n=17)* | | | | | | | | | |
| Adametz et al., 2017 [89] | Germany | 100 |  | Community (Adolescents, Females) | To determine the long-term effects of the primary prevention program PriMa (Primary prevention of anorexia nervosa in preadolescent girls) on disordered eating and body self-esteem from childhood to young adulthood. | | Longitudinal (Follow-up: 8 years) | Disordered eating behaviours, body self-esteem, body mass index | |
| Austin et al., 2012 [87] | USA | 16,369 |  | Community (Adolescents, Females) | To discover the effects of disordered weight control behaviour (DWCB) when implemented by schools under dissemination conditions | | Quasi-experimental (intervention) (Follow-up: 3 years) | Disordered weight control behaviour | |
| Berger et al., 2014 [90] | Germany | 533 |  | Community (Children, Both sexes) | To assess the effects of a German school-based primary prevention program (“Torera”) for seventh graders | | Quasi-experimental (intervention) | Body self-esteem, eating behaviour, BMI | |
| Buerger et al., 2019 [92] | Germany | 1654 |  | Community (Adolescents, Both sexes ) | To develop and examine a school-based universal prevention program (‘MaiStep’) for adolescent boys and girls | | RCT (Intervention: 5 weeks; Follow-up: 12 months) | Eating disorder-related risk factors (Eating Disorder Inventory-2), cognitions and affect related to the  body (Body Shape Questionnaire, BSQ-8), and behaviours (Body Image Avoidance Questionnaire, BIAQ) | |
| Domine et al., 2009 [91] | Switzerland | 3890 |  | Community (Adolescents, Males) | To determine the characteristics specific to boys with disordered eating behaviours (DEB) and the general context in which these DEB occur | | Cross-sectional | Disordered eating behaviours | |
| Dunstan, Paxton & McLean, 2017 [96] | Australia | 200 |  | Community (Adolescents, Males) | To evaluate the co-educational body image intervention (Happy Being Me Co-educational) program's efficacy, and to identify whether girls would benefit equally when it was delivered as a universal intervention to a whole class including both boys and girls (co-educational delivery), or delivered as a selective intervention to girls only (single-sex delivery) | | Repeated Measure (with follow-up) (Follow-up: 6 months) | Body dissatisfaction; Internalization of the thin ideal; appearance comparison; self-esteem; weight-related teasing; appearance conversation; dietary restraint | |
| Jones et al., 2014 [99] | USA | 336 |  | Community (Adolescents, Females) | To describe an online program, StayingFit, which has two tracks for universal and targeted delivery and was designed to enhance healthy living skills, encourage healthy weight regulation, and improve weight/shape concerns among high school adolescents | | Quasi-experimental (Intervention: 12 sessions) | Anthropometric measures, weight and shape concerns | |
| Leme et al., 2020 [166] | Worldwide |  | 35 | Community (Adolescents, Both sexes) | To compare the impact of “energy-balance” and “shared risk factor for obesity and eating disorders” prevention programs on weight outcome changes; and (2) if the eating disorder risk factors were improved in the “shared risk factor for obesity and eating disorders” programs. | | Systematic Review/ Meta-Analysis (combined) | Body or shape satisfaction,  weight-control behaviours, weight-teasing and/or diet intake (measured via reports), PA levels, BMI | |
| Richardson & Paxton, 2010 [95] | Australia | 194 |  | Community (Adolescents, Females) | To evaluate the efficacy of a theoretically derived school-based body image intervention for young adolescent girls | | Quasi-experimental (Intervention: 3 sessions) (Follow-up: 3 months) | Intervention topic knowledge, risk  factors for body dissatisfaction, body  image, dietary restraint and self-esteem | |
| Schwartz et al., 2019 [13] | Germany |  | 23 | Community (Mixed Cohort, Both sexes) | To systematically review recent studies on the universal prevention of ED | | Systematic Review/ Meta-Analysis (combined) | Thin-ideal internalisation, eating behaviour, weight and shape concern, dietary restraints, self-esteem, body satisfaction, eating pathology | |
| Sharpe et al., 2013 [93] | England | 448 |  | Community (Adolescents, Females) | To assess the acceptability, feasibility, and efficacy of a teacher-delivered body image intervention. | | RCT (Intervention: 6 sessions; Follow-up: 3 months) | Body esteem, presence of binge eating, compensatory behaviours, thin-ideal internalisation, appearance conversations, depressive symptoms | |
| Taylor et al., 2016 [84] | USA | 206 |  | Community (Adult, Females) | To evaluate an indicated online eating disorder (ED) intervention, designed to reduce ED and comorbid pathology | | RCT (Intervention: 10 weeks; Follow-up: 2 years) | Rate of ED onset, disordered eating behaviour, weight concern, coping skills, binge drinking episodes | |
| Warschburger & Zitzmann, 2018 [94] | Germany | 1112 |  | Community (Adolescents, Both sexes) | To evaluate the efficacy of the POPS-program (POtsdam Prevention at Schools), a universal school-based eating disorder prevention program for adolescents | | RCT (Intervention: 9 weeks; Follow-up: 1 year) | Indicators of disordered eating and relevant risk factors for eating disorders (body dissatisfaction, internalization of the thin ideal, perceived media pressure, perfectionism, emotional element of exercise, social comparison, and perceived teasing) | |
| Wick et al., 2011 [88] | Germany | 1553 |  | Community (Children, Young females) | To assess the effect of a developed German school-based intervention program (‘PriMa’) for the primary prevention of AN in preadolescent girls | | Quasi-experimental (Intervention: 9 sessions; Follow-up: 3 months) | Body self-esteem, eating behaviours, knowledge about danger of AN | |
| Wilksch et al., 2013 [126] | Australia | 20 |  | Outpatient (Young Adult, Females) | To evaluate the effectiveness of a pilot programme in enhancing protective factors for eating disorders in young girls with type 1 diabetes (T1D). | | Quasi-experimental (Intervention: 2 sessions; Follow-up: 1 month) | Media literacy, perfectionism, self-esteem | |
| Wilksch et al., 2016 [98] | Australia | 1316 |  | Community (Adolescents, Both sexes) | To investigate if baseline shape and weight concern (SWC) moderated outcomes in Prevention Across the Spectrum, a randomized-controlled trial (RCT) of 3 school-based programs aimed at reducing eating disorder and obesity risk factors. | | RCT (Intervention: 3 sessions; Follow-up: 12 months) | Weight and shape concern, eating concern, dieting, body dissatisfaction, media internalisation, perceived pressure, depression, weight-related peer teasing, perfectionism, regular eating, screen time, physical activity | |
| Wilksch, O'Shea & Wade, 2018 [100] | Australia, New Zealand | 87 |  | Community (Young Adult, Females) | To investigate the efficacy of MS-T about clinical (or diagnostic) outcomes—namely prevention effects (outcomes for those who did not meet ED diagnosis at baseline) and treatment effects (outcomes for those who met ED diagnosis at baseline). | | RCT (Intervention: 9 weeks; Follow-up: 12 months) | ED onset, ED remission | |

**Table 2:** Studies included in the rapid review categorised by early intervention programs

| **Author** | **Country** | **N participants** | **N studies** | **Population** | **Aim** | **Design** | **Outcome measure** |
| --- | --- | --- | --- | --- | --- | --- | --- |
| **Early Intervention (*n*=35)** | | | | | | | |
| Ali et al., 2017 [134] | Australia |  | 13 | Community (Mixed Cohort, Both sexes) | To systematically review the literature on perceived barriers and facilitators of help-seeking for eating disorders. | Review (Systematic) | Stigma and shame, Denial/failure to perceive severity of the illness/lack of awareness, Practical barriers, Fear of losing control/fear of change/low motivation to change, Negative attitudes towards treatment/perceived insufficiency of professional help, Lack of encouragement/support from others, lack of understanding from others, Knowledge of resources/not knowing how to ask/access help Accessibility to help/support/treatment Self-sufficiency, Inability of others to provide help Mental health/eating disorder literacy Use of other resources Previous negative experience with treatment Not wanting to hurt others/concern for others Comorbidity (depression/anxiety) |
| Austin et al., 2021 [21] | England | 278 |  | Community (Mixed Cohort, Young males) | To assess the scalability of First Episode Rapid Early Intervention for Eating Disorders (FREED) | Quasi-experimental (intervention) (Follow-up: 1 year) | ED symptoms, mood, service utilization and cost |
| Becker et al., 2009 [177] | USA |  | N/A | Community (Adult, Both sexes) | To review the principles of community-partnership research (CPR) and explores why CPR might improve distribution of psychological ESIs. | Review (Other) | Dissemination and use of Empirically Supported Interventions (ESI) in clinical setting |
| Beintner, Jacobi & Taylor, 2012 [152] | USA and Germany | 990 | 10 | Community (Mixed Cohort, Young females) | To integrate, using meta‐ analytic techniques, the effects of StudentBodies^TM^ at US and German sites and to examine the role of participants’ risk status as a potential moderator in an exploratory analysis. | Meta-Analysis | Drive for thinness, bulimia, body dissatisfaction, restraint, weight concern, shape concern, weight concern scale, Body shape Questionnaire, knowledge test |
| Bohrer et al., 2017 [159] | USA | 595 |  | Community (Adult, Both sexes) | To identify variables most predictive of treatment seeking in a nationally representative sample of adults with EDs. | Modelling (Statistical) | Predictor of treatment seeking |
| Coffino, Udo & Grilo, 2019 [160] | USA | 36,309 |  | Community (Adult, Both sexes) | To investigate, in a nationally representative sample of US adults, the prevalence of help- seeking in individuals with Diagnostic and Statistical Manual of Mental Disorders, Fifth Edition (DSM-5) eating disorders (EDs) and to examine sex and ethnic/racial differences. | Cross-Sectional | Help seeking, sex, ethnic or racial differences, ED type |
| Denison-Day et al., 2018 [133] | UK | 3421 | 42 | Community (Mixed Cohort, Both sexes) | To explore the evidence for improving motivation to change in eating disorders via clinical interventions. | Review (Systematic) | Motivation to change |
| Denison-Day et al., 2019 [145] | UK | 313 |  | Community (Adult, Both sexes) | To assess the impact of MotivATE on attendance at assessment when compared with treatment-as-usual. | RCT (Intervention: 4 sessions; Follow-up: post-intervention) | Attendance at initial assessment appointment, engagement with the intervention |
| Doley et al., 2017 [135] | Australia | 5916 | 18 | Community (Mixed Cohort, Both sexes) | To describe effectiveness of intervention strategies, and identified gaps in the literature | Review (Systematic) | Measures of stigma |
| Fatt et al., 2020 [11] | Australia | 1002 |  | Community (Adolescents, Both sexes) | To provide the first population-based investigation of help-seeking correlates among adolescents with an eating disorder. | Cross-Sectional | Body image and help seeking assessment, ED status, Demographics: age, SES, migrant status, sexuality, age, BMI) |
| Fitzsimmons-Craft et al., 2019 [155] | USA | 185 |  | Community (Adult, Females) | To examine short-term (i.e., 1 month) reciprocal longitudinal relations between weight/shape concern and comorbid symptoms (i.e., depressed mood, anxiety) and behaviours (i.e., binge drinking) over the course of 24 months using cross-lagged panel models. | RCT (Intervention: 10 weeks; Follow-up: 2 years) | Weight shape concern, ED risk status, depressed mood, anxiety, binge drinking |
| Fursland et al., 2018 [139] | USA | 448 |  | Outpatient (Adult, Both sexes) | To examine the effect on waitlist length, attendance, and eating disorder symptoms, of a 75–90 min single session intervention (SSI), attended a median of 16 days after referral to a specialist eating disorders clinic. | Quasi-experimental (Intervention: 1 session) | Waitlist length, attendance and eating disorder symptom |
| Gratwick-Sarll & Bentley, 2014 [158] | Australia | 177 |  | Community (Adult, Both sexes) | To determine whether a training intervention on mental health first aid for eating disorders improved knowledge, attitudes, and behaviours towards people with eating disorders. | Quasi-experimental (Intervention: 1 session) | Participant knowledge, attitudes, and helping behaviours for eating disorders |
| Gratwick-Sarll et al., 2016 [162] | Australia | 139 |  | Community (Adolescents, Young females) | To examine self-recognition of disordered eating and factors associated with this among female adolescents with bulimic-type eating disorders recruited from a large, population-based sample | Cross-sectional | Eating disorder psychopathology, general psychological distress and functional impairment, and frequency of occurrence of specific eating disorder behaviours |
| Gumz et al., 2018 [136] | Germany | 59 |  | Community (Mixed Cohort, Young females) | To investigate the effect of a systemic public health intervention on the length of time between anorexia nervosa symptom onset and contact with the health care system as well as the initiation of treatment. | Repeated Measure (without follow-up) | Duration of untreated illness, Duration until first contact with the healthcare system |
| Hart and Jorm, 2009 [156] | Australia | 85 |  | N/A | To develop first aid guidelines, based on expert consensus, that provide members of the community with information on how to assist someone who is thought to be developing or experiencing an eating disorder. | Cross-Sectional | Endorsement/ratings of statements |
| Hart et al., 2012 [157] | Australia | Study 1 = 706, Study 2= 154 |  | Community (Mixed Cohort, Both sexes) | To evaluate the usefulness and impact of the guidelines on web users who download them. | Cross-Sectional | Provision of first aid to others, seeking help for self and success rate |
| Hart, Jorm & Paxton, 2012 [7] | Australia | 73 |  | Community (Adult, Both sexes) | To examine whether specific training in mental health first aid for eating disorders was effective in changing knowledge, attitudes, and behaviours towards people with eating disorders. | Quasi-experimental (brief intervention; Follow-up: 1 month) | Problem recognition item within the MHLQ-B and the FAKT |
| Hötzel et al., 2014 [148] | Germany | 212 |  | Community (Adult, Females) | To investigate the effects of an internet-based program (‘ESS-KIMO’) to enhance motivation to change in eating disorders. | RCT (Intervention & Follow-up: 6 weeks) | Motivation to change, Eating Disorder Examination Questionnaire (EDE-Q), Stages of Change Questionnaire for Eating Disorders (SOCQ-ED). Self-efficacy Scale, Rosenberg self-esteem scale, pros, and cons of eating disorders scale |
| Jacobi et al., 2011 [153] | USA | 236 |  | Community (Adult, Females) | (1) To identify risk factors and their interaction for ED onset in a high-risk sample of college- age females using the methods developed by Kraemer et al. (1997, 2001) and (2) to determine the most potent risk factors for ED onset (including sensitivity, specificity, and optimal cut-offs) in a high-risk sample. | Longitudinal (3 years) | Time to onset of a subthreshold or full ED |
| Jacobi et al., 2018 [151] | Germany | 66 |  | Community (Adolescents, Females) | To evaluate the efficacy of an indicated, parent-based, Web-based preventive program Eltern als Therapeuten (E@T) in reducing risk factors and symptoms of AN. | RCT (Intervention: 6 weeks; Follow-up: 1 year) | Eating Disorder Inventory; Eating Disorder Examination. |
| Jones et al., 2012 [150] | USA and Germany | 46 |  | Community (Adolescents, Females) | To explore whether potential risk factors for anorexia nervosa (AN) can be modified by a family-based Internet- facilitated intervention and examines the feasibility, acceptability, and short-term efficacy of the Parents Act Now programme in the USA and Germany. | Quasi-experimental (Intervention: 6 weeks; Follow-up: 1 year) | Restraint, eating concern, weight concern, shape concern, drive for thinness, perfectionism |
| Kass et al., 2014 [154] | USA | 302 |  | Community (Mixed Cohort, Both sexes) | To investigate whether the guided discussion group improves program efficacy in reducing weight/shape concerns in females at high risk for an eating disorder | RCT (Intervention & Follow-up: 8 weeks) | Body Mass Index; Weight Concerns Scale; Beck Depression Inventory; Willingness to Improve Body Image; Willingness to Improve Emotion Regulation; Eating Disorder Examination Questionnaire. |
| Kindermann et al., 2017 [144] | Germany | 394 |  | Community (Young Adult, Both sexes) | To analyse associations between ED-related symptoms and utilization of the individualized program ProYouth both between- and within-persons, aiming to investigate whether participants adapt utilization intensity to their current needs. | Cross-Sectional | ED related symptoms, participation in chat and forum, page visits |
| Mazzeo et al., 2016 [143] | USA | 45 |  | Community (Adolescents, Females) | To examine satisfaction, feasibility, and preliminary outcomes of a binge eating intervention for ethnically diverse adolescent girls. | RCT (Intervention: 8-12 weeks; Follow-up: 3 months)  (Incentive: yes) | EDE-Q, EES-C, EAH: Eating concern Shape concern Weight concern Restraint, Anxiety, anger, and frustration Depressive symptoms Feeling unsettled, negative affect, external, fatigue |
| McClelland et al., 2018 [137] | UK | 75 |  | Community (Adult, Both sexes) | To assess the impact of FREED (First Episode Rapid Early Intervention for Eating Disorders [ED]), a novel transdiagnostic service for emerging adults with recent ED onset, on clinical outcomes. | Cross-Sectional | EDE-Q, Clinical outcomes in routine evaluation, DASS-21, Work and social adjustment scale, Levels of expressed emotions scale, CIA (patients), Accommodation and Enabling scale (AESED) |
| McClelland et al., 2020 [161] | Worldwide |  | 22 | Community (Children & Adolescents, Both sexes) | To explore which symptoms (both ED and other psychiatric disorder-related) exist prior to the onset of an ED and whether there any prospective associations between these symptomatologies. | Review (Systematic) | Symptom trajectory preceding illness onset |
| McLean, Paxton & Wertheim, 2011 [146] | Australia | 101 |  | Community (Adult, Females) | To examine the outcome of a body image and disordered eating intervention for midlife females. | RCT (Intervention: 8 weeks; Follow-up: 6 months) | Body dissatisfaction Weight and shape concern Body image avoidance Appearance comparison Internalization Appearance importance Cognitive reappraisal Self-care physical Self-care attitude Restraint External eating Emotional eating Kessler distress |
| Mond et al., 2010 [175] | Australia | 756 |  | Community (Adult, Females) | To compare attitudes and beliefs concerning the nature and treatment of bulimia nervosa (BN) among young adult females | Cross-Sectional | mental health literacy survey, EDE-Q (restraint, eating, weight shape concern) |
| Neubauer et al., 2014 [20] | Germany | 140 |  | Community (Mixed Cohort, Young females) | To examine paths to first treatment and the duration of untreated illness in 140 anorexia nervosa patients using validated question- naires and a clinical interview. | Cross-Sectional | Socioeconomic variables, ED diagnosis, onset and duration of untreated illness, ED pathology, current depressive symptom |
| Nicholls and Yi, 2012 [132] | UK |  | N/A | Community (Mixed Cohort, Both sexes) | To synthesize findings from five key theoretical domains, and present pilot data from a phase-specific early intervention for new onset EDs in young people. | Review (Narrative) | Parental concern about changes in eating behaviour and/or weight loss |
| Ruwaard et al., 2013 [149] | Netherlands | 105 |  | Community (Mixed Cohort, Both sexes) | To assess the efficacy of a new online CBT of bulimic symptoms | RCT (Intervention: 20 weeks; Follow-up: 1 year) | Eating Disorder Examination Questionnaire (EDE-Q), and Body attitudes test |
| Schleider & Weisz, 2017 [138] | Worldwide | 10,508 | 50 | Community (Young adult, Both sexes) | To assess the effects of single-session interventions for youth psychiatric problems | Meta-analysis | Youth problem type (depression, anxiety, substance abuse), youth problem severity |
| Shomaker et al., 2017 [140] | USA | 29 |  | Community (Children, Both sexes) | To evaluate feasibility and acceptability of a preventive family-based interpersonal psychotherapy (FB-IPT) program, and to compare FB-IPT to family-based health education (FB-HE) for evaluating changes in children’s psychosocial functioning, LOC-eating, and body mass. | RCT (Intervention: 12 weeks; Follow-up: 1 year) (Incentive: yes) | Social problems, depression, anxiety, BMI, disordered eating, body fat |
| Tanofsky-Kraff et al., 2014 [141] | USA | 112 |  | Community (Adolescents, Females) | To determine whether an adapted interpersonal psychotherapy prevention program is more efficacious for reducing excess weight gain and worsening disordered eating than health education in adolescent girls at high risk of obesity and eating disorders. | RCT (Intervention: 12 weeks; Follow-up: 1 year) | Weight maintenance, BMI, and percentage of adiposity |
